# Phenothiazine and semi-cyanine based colorimetric and fluorescent probes for detection of sulfites in solutions and in living cells†

**DOI:** 10.1039/d1ra06868g

**Published:** 2021-10-26

**Authors:** Hong-Wei Chen, Hong-Cheng Xia, O. A. Hakeim, Qin-Hua Song

**Affiliations:** Department of Chemistry, University of Science and Technology of China Hefei 230026 P. R. China qhsong@ustc.edu.cn xiahc@mail.ustc.edu.cn; School of Pharmacy, Xinxiang Medical University Xinxiang Henan 453003 P. R. China; National Research Centre, Textile Research Division Tahrir St., Dokki Cairo Egypt ohakeim@yahoo.com

## Abstract

Four hemicyanine probes for selectively detecting sulfites (HSO_3_^−^/SO_3_^2−^) have been constructed by the condensation reaction of 7-substituted (CN, Br, H and OH) phenothiazine aldehyde with 1-ethyl-2,3,3-trimethylindolium iodide. All four probes show a fast and sensitive response to HSO_3_^−^/SO_3_^2−^*via* a Michael addition, with a detection limit lower than 40 nM based on monitoring their UV/vis absorption changes. Although all four probes display an increase in fluorescence when responding to HSO_3_^−^/SO_3_^2−^, the increment is larger for the probe with an electron-withdrawing group than the probe with an electron-donating group, except for Br. Thus, among four probes the 7-cyano probe (PI-CN) possesses the largest fluorescent response to HSO_3_^−^/SO_3_^2−^, and the lowest detection limit (7.5 nM). More expediently and easily, a film and a test paper with PI-CN have been prepared to detect HSO_3_^−^/SO_3_^2−^ in a sample aqueous solution selectively. Finally, the detection of HSO_3_^−^/SO_3_^2−^ by PI-CN in biological environments has been demonstrated by cell imaging.

## Abstract 2

Four 7-substituted phenothiazine hemicyanines display a substituent effect on the fluorescence response toward sulfites. The CN-substituted probe exhibits the best sensing behavior.

## Graphical abstract

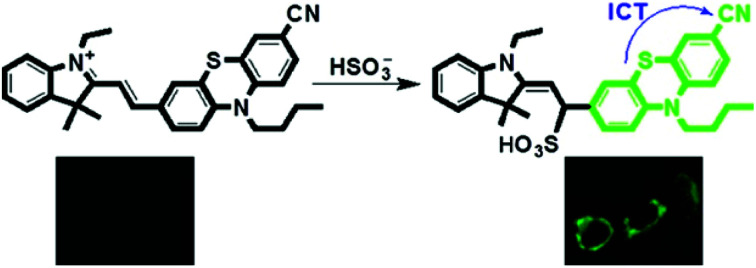

## Introduction

Sulfur dioxide (SO_2_) is both a main atmospheric pollutant and a valuable commercial reagent, and human exposure to SO_2_ has become increasingly widespread due to the combustion of fossil fuels and industrial manufacture, for example paper pulp manufacturing and metal processing. More and more medical studies have confirmed that exposure to SO_2_ may not only cause respiratory responses,^1^ but also lead to lung cancer, cardiovascular diseases, and some neurological diseases, such as migraine headaches and brain cancer.^2^ SO_2_ dissolves in water to form a pH dependent equilibrium mixture between bisulfite and sulfite (HSO_3_^−^/SO_3_^2−^) with a molar ratio of about 3 : 1 in neutral aqueous solution.

Bisulfite and sulfite are widely used as preservatives for food and beverages to prevent oxidation and bacterial growth.^3^ However, sulfite is toxic in high doses, which is associated with allergic reaction and food intolerance symptoms, such as mild to severe skin allergy, asthma, or gastrointestinal diseases.^4^ Hence, the Joint FAO/WHO Expert Committee on Food Additives has issued that an acceptable daily intake should be lower than 0.7 mg kg^−1^ of body weight; the U.S. Food and Drug Administration (FDA) has required the labeling of products containing no more than 10 ppm (125 μM) sulfite in foods or beverages.^5^

Endogenous HSO_3_^−^ and SO_3_^2−^ can be metabolically generated from thiol-containing amino acids, such as cysteine and glutathione.^6^ The studies have showed that HSO_3_^−^/SO_3_^2−^ have an endothelium-dependent vasorelaxing effect at low concentrations (<450 μM) and also function as messengers in cardiovascular systems.^7^ For this reason, the development of new detection methods for HSO_3_^−^/SO_3_^2−^ is important for environmental security and human health. Due to the advantages of simplicity, sensitivity, nontoxicity, and ease of operation, fluorescent probes have been recognized as efficient molecular tools for visualizing anions in living systems.^8^ In recent years, some excellent fluorescent probes have been reported by various effective reactions of HSO_3_^−^/SO_3_^2−^ including nucleophilic addition to an aldehyde,^9^ mediated levulinate cleavage,^10^ nucleophilic addition to an unsaturated bond,^11^ and others.^12^ However, there are still some drawbacks in reported probes such as poor selectivity over biothiols or H_2_S for most aldehyde- or levulinate-based fluorescent probes, long response time (>5 min for most probes), high detection limits (>1 μM for half of probes). Hence, it is still a challenge to develop more convenient and quick response probes for HSO_3_^−^/SO_3_^2−^. In this work, we prepared four 7-substituted phenothiazine-hemicyanine probes (PI-CN, PI-Br, PI-H and PI-OH), which can detect HSO_3_^−^/SO_3_^2−^ in solutions fast and sensitively. Among the four probes, PI-CN displays the best response performance to HSO_3_^−^/SO_3_^2−^. Additionally, further application of film or test paper detection based on PI-CN and bioimaging in living cells were performed.

## Experimental

### Reagents and instrumentation

All the chemicals for synthesis were purchased from commercial suppliers and were used as received without further purification. ^1^H and ^13^C NMR spectra were measured in CDCl_3_ or DMSO-d_6_ with a Bruker AV spectrometer operating at 400 MHz and 100 MHz, respectively and chemical shifts were reported in ppm using tetramethylsilane (TMS) as the internal standard. Mass spectra were obtained with a Thermo LTQ Orbitrap mass spectrometer. UV-vis absorption and fluorescence emission spectra were recorded with a Shimadzu UV-2450 UV/vis spectrometer and a Shimadzu RF-5301pc luminescence spectrometer, respectively.

### Preparation of sample solutions

Sample solutions for the measurement were EtOH/PBS (v/v 1 : 3) solvent mixtures, the concentration of PBS is 10 mM. All concentrations of the probes for test were 15 μM in above solution. Water for sample solution preparation was purified with a Millipore water system. All pH values were measured on a MQK PHS-3C pH meter.

PI-CN (0.1 mg) was dissolved in 1.8 mL gel buffer (water was added to 2 M (24.23 g) Tris. Base and 0.2% (0.2 g) SDS to final volume of 100 mL), 1.1 mL 49.5% acylamide with 3% diacylamide (water was added to 48 g acylamide and 1.5 g bisacylamide to final volume of 100 mL), 50 μL 10% APS and 0.6 mL of 80% glycerol, then 10 μL tetramethylethylenediamine (TEMED) was added to form the film containing PI-CN.

### Cell culture and cell images

HeLa cells were seeded on the coverslips in 24-well plates at a density of 50 000 cells per well and incubated in a humidified 5% CO_2_ atmosphere for 24 h with the complete Dulbecco's modified eagle's medium (DMEM) containing 10% fetal calf serum at 37 °C. 10 μL of DMSO solution of PI-CN (1 mM) was added to a well to give concentration of 10 μM and volume ratio of DMSO/culture medium is 1 : 100. After incubation for 30 min, and then the cells were washed three times with PBS buffer after culture medium was removed. The cells were further treated with 0.1 mM NaHSO_3_ for 30 min, then removed the culture medium and washed three times with PBS buffer. The cellular localization was visualized under a laser scanning confocal microscope (LSM 710 Meta, Carl Zeiss Inc., Thornwood, NY). The green fluorescence of cells was collected with 476–526 nm channel under excitation at 405 nm.

### Synthesis of 7-bromo-10-butyl-10*H*-phenothiazine-3-carbaldehyde (2)

A round-bottom flask was charged with compound 1 (1.5 g, 5.3 mmol) and 5 mL of THF. A solution of *N*-bromosuccinimide (0.95 g, 5.3 mmol) in 3 mL of THF was added to the stirred mixture dropwise. The mixture was stirred for 12 h at room temperature in the absence of light and oxygen. Then 40 mL water and 40 mL DCM was added. The organic phase was separated and the washed with water (20 mL) three times. The organic phases were dried with anhydrous magnesium sulphate. Then the solvent was removed under reduced pressure. Purification by column chromatography on silica (PE/EA, v/v 40 : 1) to afford compound 2 (1.26 g, 66%) as yellow oil. *R*_f_ = 0.4 (PE/EA 10 : 1); ^1^H NMR (400 MHz, CDCl_3_, 25 °C, TMS): *δ* = 9.80 (s, 1H, CHO), 7.64 (dd, *J* = 8.4 Hz, *J* = 1.7 Hz, 1H, Ar-H), 7.57 (d, *J* = 1.7 Hz, 1H, Ar-H), 7.22–7.26 (m, 2H, Ar-H), 6.90 (d, *J* = 8.4 Hz, 1H, Ar-H), 6.72 (t, *J* = 8.6 Hz, 1H, Ar-H), 3.86 (t, *J* = 7.1 Hz, 2H, N–CH_2_), 1.73–1.81 (m, 2H, CH_2_), 1.41–1.50 (m, 2H, CH_2_), 0.94 (t, *J* = 7.4 Hz, 3H, CH_3_) ppm.

### Synthesis of 10-butyl-7-formyl-10*H*-phenothiazine-3-carbonitrile (3)

Under a nitrogen atmosphere a mixture of compound 2 (500 mg, 1.38 mmol), sodium carbonate (146 mg, 1.38 mmol), K_4_[Fe(CN)_6_] (114 mg, 0.35 mmol), dppf (15.3 mg, 0.03 mmol), and palladium(ii) acetate (3.1 mg, 0.014 mmol) in 5 mL of *N*-methyl-2-pyrrolidone was heated at 120 °C for 17 h, and cooling to room temperature the oversaturated aqueous solution of Na_2_SO_3_ (10 mL) was added and the organic phase separated. The aqueous layer was extracted three times with 10 mL dichloromethane. The combined organic layers was dried with anhydrous sodium sulfate and the solvent was removed *in vacuo*. The residue was chromatographed on silica gel (PE/EA, v/v 30 : 1) to yield compound 3 (306 mg, 72%) as a yellow solid. *R*_f_ = 0.39 (PE/EA 4 : 1); ^1^H NMR (400 MHz, CDCl_3_, 25 °C, TMS): *δ* = 9.82 (s, 1H, CHO), 7.68 (dd, *J* = 8.4 Hz, *J* = 1.8 Hz, 1H, Ar-H), 7.57 (d, *J* = 1.8 Hz, 1H, Ar-H), 7.44 (dd, *J* = 1.9 Hz, 1H, Ar-H), 7.32 (d, *J* = 1.8 Hz, 1H, Ar-H), 6.96 (d, *J* = 8.5 Hz, *J* = 8.4 Hz, 1H, Ar-H), 6.89 (d, *J* = 8.4 Hz, 1H, Ar-H), 3.91 (t, *J* = 7.2 Hz, 2H, N–CH_2_), 1.76–1.83 (m, 2H, CH_2_), 1.43–1.52 (m, 2H, CH_2_), 0.97 (t, 3H, *J* = 7.4 Hz, CH_3_) ppm. ^13^C NMR (100 MHz, CDCl_3_, 25 °C, TMS) *δ* = 198.8, 149.0, 147.6, 132.1, 131.9, 130.5, 130.3, 128.5, 125.1, 124.1, 118.4, 115.8, 115.7, 106.6, 48.0, 28.6, 20.0, 13.7 ppm. HRMS (ESI) *m*/*z* calcd for C_18_H_16_N_2_OS + H^+^: 309.1062 ([M + H^+^]), found: 309.1055.

### Synthesis of 10-butyl-7-(4,4,5,5-tetramethyl-1,3,2-dioxaborolan-2-yl)-10*H*-phenothiazine-3-carbaldehyde (4)

Under a nitrogen atmosphere, a mixture of compound 2 (402 mg, 1.1 mmol), bis(pinacolato)diboron (422 mg, 1.66 mmol), Pd(dppf)_2_Cl_2_ (32 mg, 0.044 mmol), potassium carbonate (326 mg, 3.32 mmol) in 5 mL of 1,4-dioxane was heated at 85 °C for 12 h. After cooling to room temperature, water (10 mL) and ethyl acetate (20 mL) was added, the organic phase was separated. The organic layer was washed with water twice (10 mL). Then the organic layers was dried with anhydrous sodium sulfate and the solvent was removed *in vacuo*. The residue was chromatographed on silica gel (PE/EA, v/v 20 : 1) to yield compound 4 (227 mg, 50%) as a yellow oil. *R*_f_ = 0.52 (PE/EA 10 : 1); ^1^H NMR (400 MHz, CDCl_3_ 25 °C, TMS): *δ* = 9.79 (s, 1H, CHO), 7.62 (dd, *J* = 8.4 Hz, *J* = 2 Hz, 1H, Ar-H), 7.59 (dd, *J* = 8.1 Hz, *J* = 1.4 Hz, 1H, Ar-H), 7.56 (d, *J* = 1.9 Hz, 1H, Ar-H), 7.53 (d, *J* = 1.4 Hz, 1H, Ar-H), 6.89 (d, *J* = 8.4 Hz, 1H, Ar-H), 6.86 (d, *J* = 8.1 Hz, 1H, Ar-H), 3.90 (t, *J* = 7.1 Hz, 2H, N–CH_2_), 1.75–1.82 (m, 2H, CH_2_), 1.45–1.51 (m, 2H, CH_2_), 1.33 (s, 12H, CH_3_), 0.94 (t, 3H, *J* = 7.4 Hz, CH_3_) ppm. ^13^C NMR (100 MHz, CDCl_3_, 25 °C, TMS): *δ* = 190.1, 150.3, 146.0, 134.4, 133.9, 131.2, 129.9, 128.4, 125.2, 123.0, 115.3, 114.9, 83.9, 47.7, 28.8, 25.0, 24.8, 20.0, 13.7 ppm. HRMS (ESI) *m*/*z* calcd for C_23_H_28_BNO_3_S + H^+^: 410.1961 ([M + H^+^]), found: 410.1962.

### Synthesis of 10-butyl-7-(4,4,5,5-tetramethyl-1,3,2-dioxaborolan-2-yl)-10*H*-phenothiazine-3-carbaldehyde (5)

Batch-wised *m*-chloroperoxybenzoic acid (*m*-CPBA) (2.9 mmol) was added to compound 4 (1.2 g, 2.9 mmol) in a H_2_O/EtOH (v/v 1 : 2) solution (2 mL) in round-bottom at room temperature, and stirred overnight at room temperature. 0.1 M aqueous sodium bicarbonate (5 mL) was added to the mixture. Then the reaction mixture was extracted with EA (20 mL). The organic layer was washed with water (10 mL) and brine (10 mL), dried over with anhydrous magnesium sulfate. The crude product was purified by column chromatography on silica gel (PE/EA, v/v 40 : 1) to afford compound 5 (370 mg, 42%) as a yellow oil. *R*_f_ = 0.37 (PE/EA 6 : 1); ^1^H NMR (400 MHz, DMSO-d_6_, 25 °C, TMS): *δ* = 9.75 (s, 1H, CHO), 9.37 (s, 1H, OH), 7.66–7.69 (m, *J* = 8.4 Hz, 1H, Ar-H), 7.54–7.56 (m, 1H Ar-H), 7.05–7.10 (m, 1H, Ar-H), 6.88–6.91 (m, 1H, Ar-H), 6.63 (dd, *J* = 8.8 Hz, *J* = 2.8 Hz, 1H, Ar-H), 6.58 (d, *J* = 2.8 Hz, 1H, Ar-H), 3.90 (t, *J* = 6.5 Hz, 2H, N–CH_2_), 1.60–1.66 (m, 2H, CH_2_), 1.33–1.41 (m, 2H, CH_2_), 0.86 (t, *J* = 7.4 Hz, 3H, CH_3_) ppm. ^13^C NMR (100 MHz, DMSO-d_6_, 25 °C, TMS): *δ* = 190.8, 154.2, 151.2, 135.0, 130.7, 130.6, 128.2, 124.0, 123.3, 117.8, 115.3, 114.8, 114.2, 47.2, 28.7, 19.8, 14.1 ppm. HRMS (ESI) *m*/*z* calcd for C_17_H_17_NO_2_S + H^+^: 300.1058 ([M + H^+^]), found 300.1053.

### Synthesis of probes

A mixture of aldehyde and 1-ethyl-2,3,3-trimethyl-3*H*-indol-1-ium iodide (1 mmol, 315 mg) was dissolved in EtOH (5 mL). The reaction mixture was stirred for 12 h at 80 °C under an N_2_ atmosphere. The mixture was cooled to room temperature, solvent was evaporated *in vacuo* and compound was purified by silica gel using DCM : MeOH (v/v 25 : 1) as the eluent. Product probe was obtained as dark purple solid.

### Synthesis of (*E*)-2-(2-(10-butyl-7-cyano-10*H*-phenothiazin-3-yl)vinyl)-1-ethyl-3,3-dimethyl-3*H*-indol-1-ium (PI-CN)

According to above procedure, the reaction of compound 3 (1 mmol, 308 mg) and 1-ethyl-2,3,3-trimethyl-3*H*-indol-1-ium iodide (1 mmol, 315 mg) afforded PI-CN (284 mg, 47%) as a dark purple solid. Melting points: 142.6–145.8 °C, *R*_f_ = 0.36 (DCM/MeOH 20 : 1). ^1^H NMR (400 MHz, CDCl_3_, 25 °C, TMS): *δ* = 8.74 (d, *J* = 8.6 Hz, 1H, Ar-H), 8.06 (d, *J* = 15.8 Hz, 1H, =CH), 7.92 (d, *J* = 15.8 Hz, 1H, =CH), 7.53–7.60 (m, 5H, Ar-H), 7.43 (dd, *J* = 8.5 Hz, *J* = 1.9 Hz, 1H, Ar-H), 7.29 (d, *J* = 1.8 Hz, 1H, Ar-H), 7.08 (d, *J* = 8.8 Hz, 1H, Ar-H), 6.90 (t, *J* = 8.5 Hz, 1H, Ar-H), 5.09 (q, *J* = 7.4 Hz, 2H, N–CH_2_), 3.92 (t, *J* = 7.2 Hz, 2H, N–CH_2_), 1.83 (s, 6H, CH_3_), 1.75–1.83 (m, 2H, CH_2_), 1.63 (t, *J* = 7.2 Hz, 3H, CH_3_), 1.44–1.49 (m, 2H, CH_2_), 0.96 (t, *J* = 7.4 Hz, 3H, CH_3_) ppm. ^13^C NMR (100 MHz, DMSO-d_6_, 25 °C, TMS): 181.3, 152.8, 148.1, 147.3, 144.3, 140.9, 133.1, 133.0, 130.9, 130.4, 129.6, 129.6, 128.9, 123.8, 123.6, 123.3, 118.9, 117.4, 117.0, 115.3, 110.9, 106.0, 52.5, 47.5, 42.3, 28.5, 26.2, 19.7, 14.2, 14.0 ppm. HRMS (ESI) *m*/*z* calcd for C_31_H_32_N_3_S^+^: 478.2317 ([M − I^−^]), found 478.2322.

### Synthesis of (*E*)-2-(2-(7-bromo-10-butyl-10*H*-phenothiazin-3-yl)vinyl)-1-ethyl-3,3-dimethyl-3*H*-indol-1-ium (PI-Br)

According to the procedure, the reaction of compound 2 (1 mmol, 362 mg) and 1-ethyl-2,3,3-trimethyl-3*H*-indol-1-ium iodide (1 mmol, 315 mg) afforded PI-Br (554 mg, 84%) as a dark purple solid. Melting points: 151.5–152.8 °C, *R*_f_ = 0.38 (DCM/MeOH 20 : 1); ^1^H NMR (400 MHz, CDCl_3_, 25 °C, TMS): *δ* = 8.63 (d, *J* = 8.3 Hz, 1H, Ar-H), 8.09 (d, *J* = 15.8 Hz, 1H, =CH), 7.77 (d, *J* = 15.8 Hz, 1H, =CH), 7.53–7.61 (m, 5H, Ar-H), 7.25 (dd, *J* = 6.6 Hz, *J* = 2.1 Hz, 1H, Ar-H), 7.16 (d, *J* = 2.0 Hz, 1H, Ar-H), 7.00 (d, *J* = 8.6 Hz, 1H, Ar-H), 6.74 (d, *J* = 8.8 Hz, 1H, Ar-H), 5.02 (q, *J* = 7.0 Hz, 2H, N–CH_2_), 3.86 (t, *J* = 7.0 Hz, 2H, N–CH_2_), 1.83 (s, 6H, CH_3_), 1.75 (m, 2H, CH_2_), 1.61 (t, *J* = 7.3 Hz, 3H, CH_3_), 1.39–1.49 (m, 2H, CH_2_), 0.94 (t, *J* = 7.4 Hz, 3H, CH_3_) ppm. ^13^C NMR (100 MHz, CDCl_3_, 25 °C, TMS): 180.3, 153.8, 150.5, 143.2, 141.8, 140.4, 133.3, 130.4, 130.3, 129.6, 129.6, 129.2, 128.4, 125.6, 123.7, 122.7, 117.4, 116.1, 116.0, 114.1, 109.5, 52.0, 48.0, 43.8, 28.7, 27.3, 19.9, 14.3, 13.7 ppm. HRMS (ESI) *m*/*z* calcd for C_30_H_32_BrN_2_S^+^: 531.1470 ([M − I^−^]), found 531.1473.

### Synthesis of (*E*)-2-(2-(10-butyl-10*H*-phenothiazin-3-yl)vinyl)-1-ethyl-3,3-dimethyl-3*H*-indol-1-ium (PI-H)

According to the procedure, the reaction of compound 1 (1 mmol, 315 mg) and 1-ethyl-2,3,3-trimethyl-3*H*-indol-1-ium iodide (1 mmol, 315 mg) afforded afford PI-H (440 mg, 76%) as a dark purple solid. Melting points: 127.1–131.4 °C, *R*_f_ = 0.39 (DCM/MeOH 20 : 1); ^1^H NMR (400 MHz, CDCl_3_, 25 °C, TMS): *δ* = 8.57 (d, *J* = 8.9 Hz, 1H, Ar-H), 8.05 (d, *J* = 15.8 Hz, 1H, =CH), 7.69 (d, *J* = 15.8 Hz, 1H, =CH), 7.51–7.56 (m, 5H, Ar-H), 7.16 (dd, *J* = 5.9 Hz, *J* = 1.5 Hz, 1H, Ar-H), 7.07 (dd, *J* = 8.7 Hz, *J* = 1.5 Hz, 1H, Ar-H), 7.01 (d, *J* = 8.7 Hz, 1H, Ar-H), 6.96 (t, *J* = 7.6 Hz, 1H, Ar-H), 6.90 (d, *J* = 8.2 Hz, 1H, Ar-H), 4.98 (q, *J* = 7.0 Hz, 2H, N–CH_2_), 3.92 (t, *J* = 7.2 Hz, 2H, N–CH_2_), 1.82 (s, 6H, CH_3_), 1.76–1.83 (m, 2H, CH_2_), 1.61 (t, *J* = 7.2 Hz, 3H, CH_3_), 1.42–1.52 (m, 2H, CH_2_), 0.96 (t, *J* = 7.4 Hz, 3H, CH_3_) ppm. ^13^C NMR (100 MHz, CDCl_3_, 25 °C, TMS): 180.0, 153.9, 151.1, 143.0, 142.4, 140.4, 133.2, 130.3, 129.6, 129.6, 129.1, 128.1, 127.7, 127.4, 124.3, 123.1, 122.7, 116.2, 115.9, 114.0, 109.0, 51.9, 48.1, 43.7, 28.8, 27.4, 20.0, 14.2, 13.8 ppm. HRMS (ESI) *m*/*z* calcd for C_30_H_33_N_2_S^+^: 453.2365 ([M − I^−^]), found 453.2367.

### Synthesis of (*E*)-2-(2-(10-butyl-7-hydroxy-10*H*-phenothiazin-3-yl)vinyl)-1-ethyl-3,3-dimethyl-3*H*-indol-1-ium (PI-OH)

According to the procedure, the reaction of compound 5 (1 mmol, 299 mg) and 1-ethyl-2,3,3-trimethyl-3*H*-indol-1-ium iodide (1 mmol, 315 mg) afforded PI-OH (417 mg, 70%) as a dark purple solid. Melting points: 147.6–149.8 °C, ^1^H NMR (400 MHz, DMSO-d_6_, 25 °C, TMS): *δ* = 9.47 (s, 1H, OH), 8.31 (d, *J* = 16.0 Hz, 1H, =CH), 8.05 (s, 1H, Ar-H), 8.02 (d, *J* = 8.9 Hz, 1H, Ar-H), 7.84–7.86 (m, 2H, Ar-H), 7.55–7.63 (m, 2H, Ar-H), 7.46 (d, *J* = 16.0 Hz, 1H, =CH), 7.07–7.14 (m, 1H, Ar-H), 6.96 (d, *J* = 8.9 Hz, 1H, Ar-H), 6.63–6.66 (m, 1H, Ar-H), 6.59 (d, *J* = 2.6 Hz, 1H, Ar-H), 3.63 (t, *J* = 6.0 Hz, 2H, N–CH_2_), 3.94 (t, *J* = 6.7 Hz, 2H, N–CH_2_), 1.76 (s, 6H, CH_3_), 1.63–1.71 (m, 2H, CH_2_), 1.40–1.44 (m, 5H, CH_2_&CH_3_), 0.89 (t, *J* = 7.4 Hz, 3H, CH_3_) ppm. ^13^C NMR (100 MHz, DMSO-d_6_, 25 °C, TMS): 180.9, 154.5, 153.3, 150.5, 144.1, 141.0, 134.4, 133.4, 129.5, 129.2, 128.8, 128.5, 123.5, 123.5, 123.1, 118.1, 115.5, 114.9, 114.9, 114.1, 109.1, 52.3, 47.6, 28.8, 26.3, 22.4, 19.7, 14.1, 14.0 ppm. HRMS (ESI) *m*/*z* calcd for C_30_H_33_N_2_OS^+^: 469.2314 ([M − I^−^]), found 469.2316.

## Results and discussion

### Synthesis of probes

**Scheme 1:**
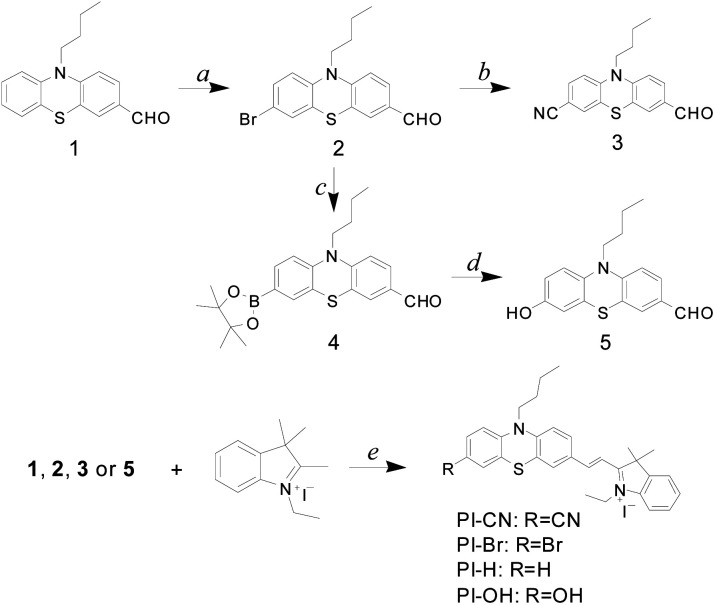
Synthetic procedure of probes: (a) NBS, THF, r.t., 12 h; (b) K_4_[Fe(CN)_6_], Pd(OAc)_2_, dppf, NMP, 120 °C, 17 h; (c) bis(pinacolato)diboron, Pd(dppf)_2_Cl_2_, K_2_CO_3_, 1,4-dioxane, 12 h; (d) *m*-CPBA, H_2_O/EtOH, r.t., 12 h; (e) EtOH, 80 °C, 12 h.

Synthetic procedure of four probes was illustrated in Scheme 1. To tune the sensing property, another three 7-substituted phenothiazine aldehydes 2, 3, and 5 were prepared with the aldehyde 1 as starting material. The condensation of four phenothiazine aldehydes (1, 2, 3 and 5) with the 1-ethyl-2,3,3-trimethylindolium iodide afforded four phenothiazine-hemicyanine compounds (PI-CN, PI-Br, PI-H and PI-OH) as HSO_3_^−^/SO_3_^2−^ probes with the yield ranging from 47% to 84%. Structures of four probes were fully characterized by ^1^H NMR, ^13^C NMR and HRMS analyses.

### Photophysical property of probes

**Fig. 1:**
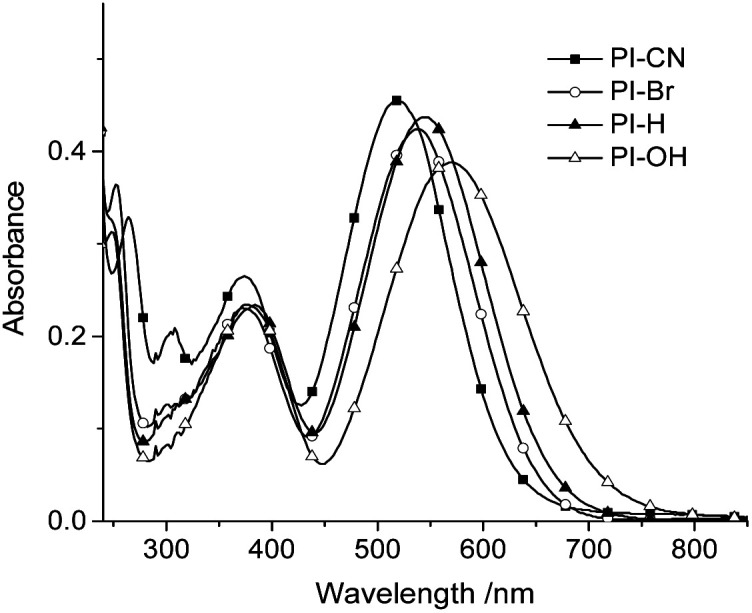
UV-vis absorption of four probes (15 μM) in EtOH/PBS (v/v 1 : 3, pH 7.4).

As optical probes for HSO_3_^−^/SO_3_^2−^, photophysical properties of four hemicyanines were first investigated. The four 7-substituents at phenothiazine cover various electronic properties, electron-withdrawing (CN), unsubstituted (H) and electron-donating (OH). Fig. 1 shows UV/vis absorption spectra of four probes in the buffer solution (EtOH/PBS v/v 1 : 3, pH 7.4). As shown in Fig. 1, the absorption maxima red shift gradually from electron-withdrawing group (CN) to electron-donating group (OH), with the corresponding values from 520 nm to 570 nm. However, all four probes have no fluorescence emission, which may ascribe to effective photoinduced *cis*–*trans* isomerism of the double bond or the formation of a TICT state. When nucleophilic addition of HSO_3_^−^ at the double bonds of probes occurs the phenothiazine moieties could emit fluorescence. The photophysical and sensing properties of four probes provided in Table S1.†

### Spectral response of probes to HSO_3_^−^/SO_3_^2−^

**Fig. 2:**
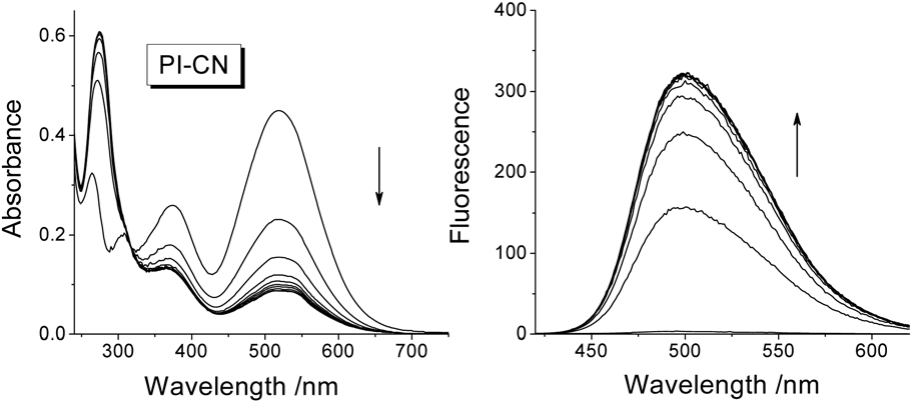
Time-dependent UV/vis absorption (left) and fluorescence spectra (right) of PI-CN (15 μM) in EtOH/PBS (v/v 1 : 3, pH 7.4) in the presence of HSO_3_^−^ (1.0 equiv.) recorded at 0–30 min, excitation at 320 nm.

After addition of 1.0 equiv. of HSO_3_^−^, the absorption spectra of 15 μM probes in EtOH/PBS (v/v 1 : 3, pH 7.4) decrease rapidly in the long-wavelength region (310–700 nm) and increase in short-wavelength region (<310 nm) (Fig. 2 for PI-CN, Fig. S1 for other three probes provided as ESI†). The four probes exhibit “turn-on” fluorescent response to HSO_3_^−^, with the most obvious for PI-CN, and the weakest for PI-Br. The latter weak fluorescence should ascribe to the heavy-atom effect of bromine. The scaffold phenothiazine is an electron rich chromophore, and modified by an electron-withdrawing group (EWG) to form a molecule with ICT character, which would reveal a longer-wavelength fluorescence emission. As shown in Fig. 2, PI-CN has the longest fluorescence emission, *λ*_max_ ∼500 nm. The probe with an electron-donating group (EDG), PI-OH, displays a weak fluorescent response. The PI-CN solution exhibit the largest fluorescence increment (>110-fold) in the presence of 1 equiv. HSO_3_^−^, among the four probes. The kinetic response time of four probes (inset of Fig. S1†) show that the sensing reaction of PI-CN could is the fastest. Hence, based on the fact that PI-CN can sense HSO_3_^−^/SO_3_^2−^ by remarkable changes by in both absorption and fluorescence, PI-CN was selected as a representative in next experiments.

Above results reveal 7-substituent has effect on the photophysical property, the sensing reactivity and the corresponding spectral response of four probes. Among them, remarkable effects are the influence of 7-substituents to the absorption spectra of probes and luminescence property of sensing products. However, there is small difference in the sensing reactivity and no observable effect on their luminescence.

**Fig. 3:**
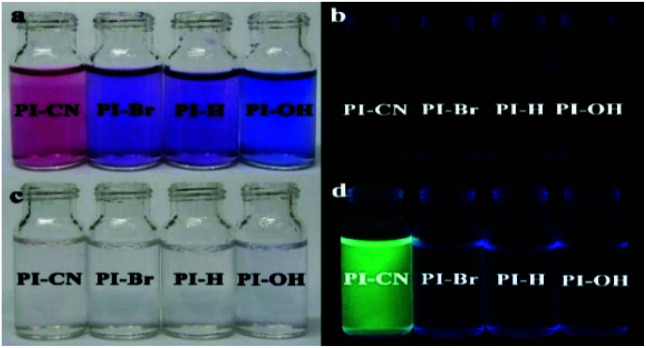
The optical (a and c) and fluorescent (b and d) photograph of probes (15 μM) without (a and b) and with (c and d) 15 μM HSO_3_^−^ in EtOH/PBS solution (v/v 1 : 3, pH 7.4).

The spectral response of four probes can be observed directly by naked eyes. The solutions of all four probes display dark purple, and no observable fluorescence emission. After addition of HSO_3_^−^, the color of all probe solutions change from red or blue to colorless (Fig. 3 left), and only the solution of PI-CN shows bright fluorescence under portable UV lamp (Fig. 3 right).

### The sensing mechanism

**Fig. 4:**
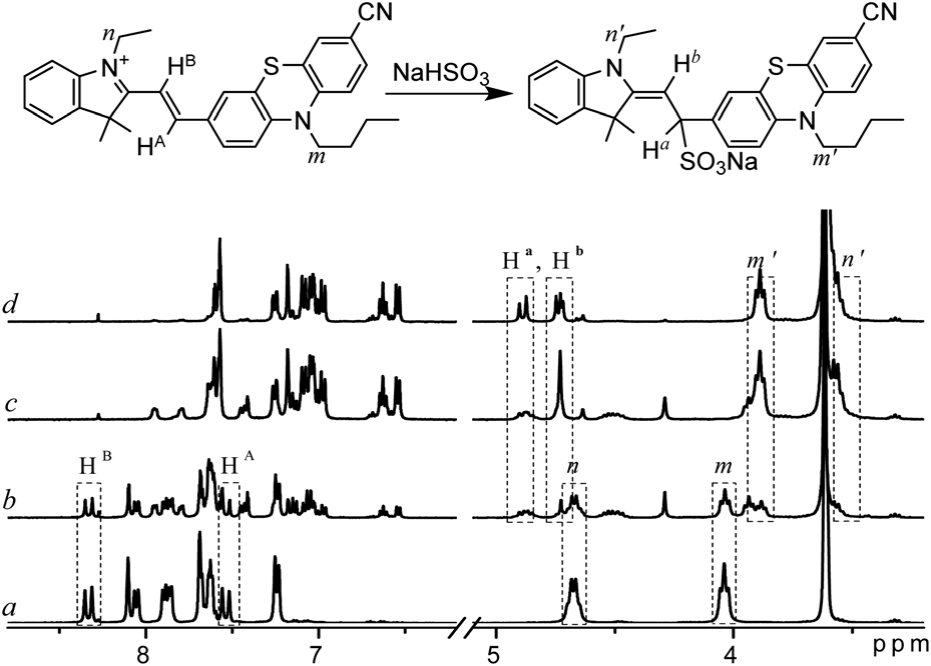
^1^H NMR spectra of PI-CN in d_6_-DMSO–D_2_O (v/v 12 : 1) before (a) and after addition of NaHSO_3_ ((b–d) amount increasing in turn).

To verify the sensing mechanism, ^1^H NMR titration of PI-CN with NaHSO_3_ was performed. As shown in Fig. 4, the chemical shifts at 8.33 ppm and 7.54 ppm were assigned to the proton H^B^ and H^A^ in PI-CN, respectively. After addition of NaHSO_3_, the proton signals of H^A^ and H^B^ disappear gradually and two groups of new peaks at 4.87 ppm and 4.72 ppm emerged which are assigned to H^b^ and H^a^. Meanwhile, other proton signals of the product appear and increase such as methylene at m′ and n′ sites.

Moreover, the formation of the adduct PI-CN-NaHSO_3_ was confirmed by high-resolution mass spectroscopy, where a dominant peak at *m*/*z* value of 582.1860 (calcd 582.1861) corresponds to [PI-CN + NaHSO_3_]^+^ provided in Fig. S2.† Therefore, the sensing reaction was confirmed to be the nucleophilic addition of the probe with HSO_3_^−^.

### Selectivity

**Fig. 5:**
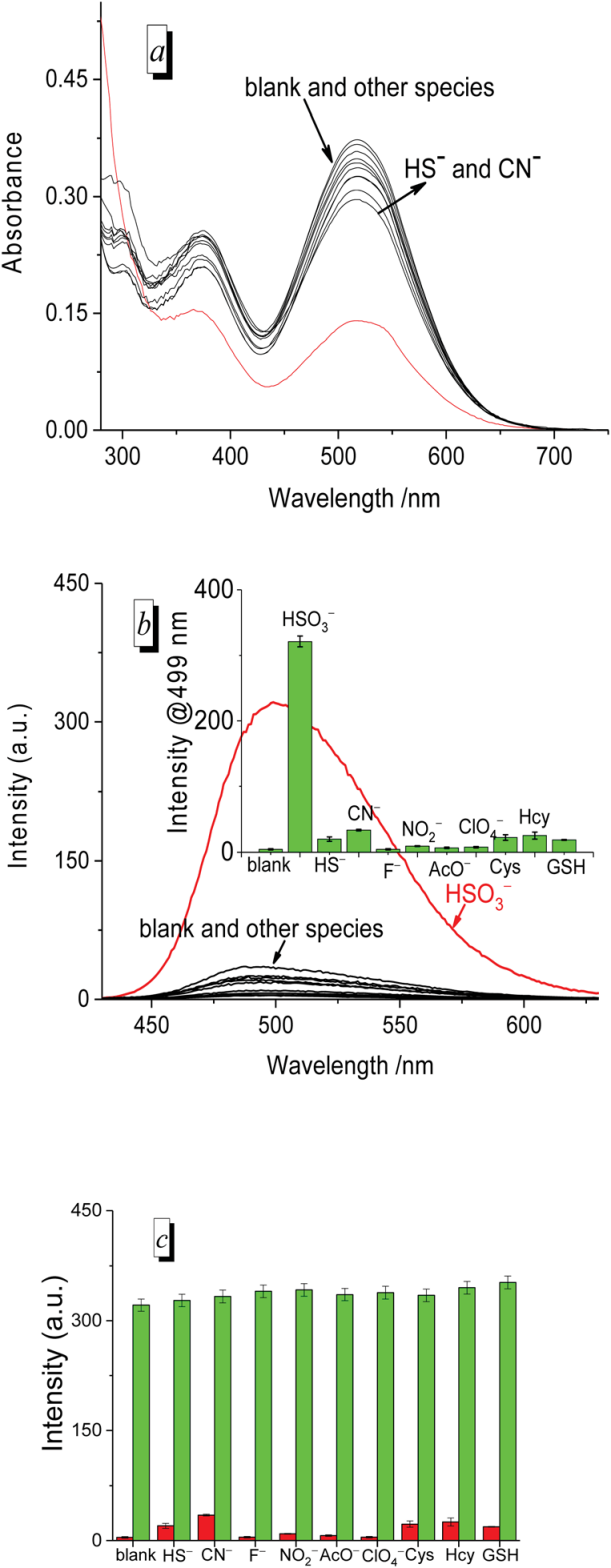
UV/vis absorption (a) and fluorescence spectra (b) of PI-CN (15 μM) in the presence of 1.0 equiv. various species (HS^−^, CN^−^, F^−^, NO_2_^−^, AcO^−^, ClO_4_^−^) and 50 equiv. of common sulphur compounds (Cys, Hcy, and GSH) in EtOH/PBS (v/v 1 : 3, pH 7.4) recorded after 12 min. Inset: fluorescence enhancement of PI-CN (15 μM) with or without various species (1.0 equiv.) at 499 nm. (c) Competing response of probe PI-CN (15 μM) towards various species (1 equiv.) in EtOH/PBS (v/v 1 : 3, pH 7.4),The red bars represent the addition of one of various species to the solution of probe; the green bars represent the fluorescence intensity of probe in the presence of various species, then added HSO_3_^−^ after 12 min.

To evaluate the selectivity of the probe for HSO_3_^−^/SO_3_^2−^, we measured the UV/vis absorption (Fig. 5a) and fluorescence spectra (Fig. 5b) of PI-CN before and after the addition of various species, respectively. The absorption and fluorescence spectra of PI-CN displayed a large change only in the presence of HSO_3_^−^, and little change for HS^−^ and CN^−^. However, other species such as F^−^, NO_2_^−^, AcO^−^, ClO_4_^−^ and biothiols caused no significant change in both UV/vis absorption and fluorescence spectra of PI-CN. The fluorescence profiles at 499 nm of the probe showed a remarkably higher selectivity for HSO_3_^−^ over the other species (inset of Fig. 5b). The fluorescence increment of PI-CN to HSO_3_^−^/SO_3_^2−^ is more than 110-fold. Moreover, the anti-interference ability of PI-CN has been studied (Fig. 5c). HSO_3_^−^ induced a great fluorescence change, whereas, other species did not result in obvious fluorescence change. The co-existence of other species didn't affect the probe's sensing behaviour to HSO_3_^−^. These results show that the tested species do not interfere with HSO_3_^−^ detection.

**Fig. 6:**
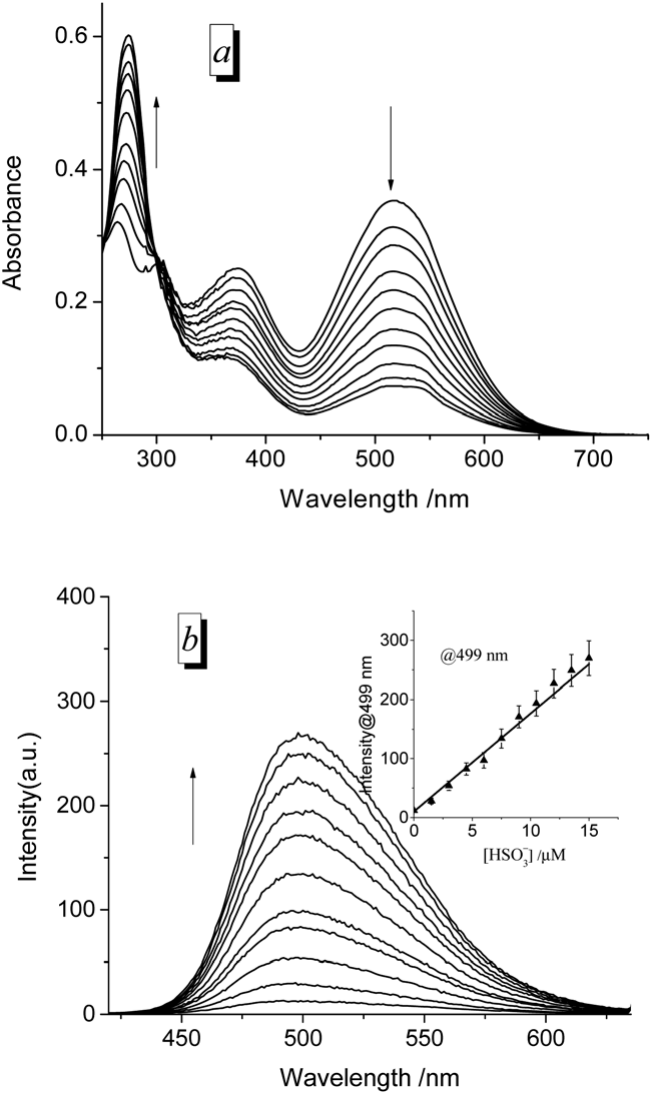
UV/vis absorption (a) and fluorescence spectra (b) of PI-CN in EtOH/PBS (v/v 1 : 3, pH 7.4) with titration of various amounts of HSO_3_^−^ (0–15 μM), incubation for 12 min, under excitation at 320 nm. Insert: linear correlation between the fluorescence intensity toward concentrations of HSO_3_^−^.

Moreover, to measure the detection limit of PI-CN, a titration experiment of HSO_3_^−^ was performed (Fig. 6). With increasing amounts of HSO_3_^−^ (0–15 μM), spectral change of PI-CN is similar to time-dependent spectral change of PI-CN after addition of 1 equiv. HSO_3_^−^. From the plot of the florescence intensity at 499 nm *vs.* the concentration of HSO_3_^−^, the detection limit of PI-CN was obtained to be 7.5 nM in terms of the signal-to-noise ratio (S/N = 3). Moreover, detection limits of the four probes to HSO_3_^−^ were measured by colorimetric analysis, 22 nM for PI-CN, 28 nM for PI-Br, 27 nM for PI-H and 37 nM for PI-OH (Fig. S3†). These values are much lower than the standard of 10 ppm (125 μM) required by the U.S. Food and Drug Administration.

**Fig. 7:**
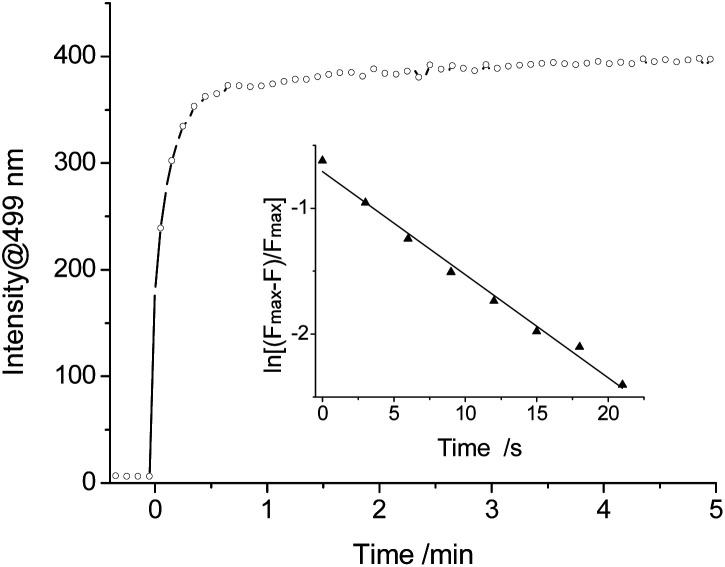
Kinetics of fluorescence enhancement rate at 499 nm for PI-CN (15 μM) with 10 equiv. HSO_3_^−^ in EtOH/PBS (v/v 1 : 3, pH 7.4) excitation at 320 nm. Inset: linear fitting of the fluorescence intensity data.

The kinetic reaction of PI-CN with NaHSO_3_ was further investigated. The time-dependent fluorescence response of PI-CN with NaHSO_3_ recorded the change of the intensity at 499 nm with time shown in Fig. 7. After addition of NaHSO_3_, the fluorescence intensity increased rapidly and reached a plateau within 1 min. The observed rate constant (*k*_obs_) of the reaction between PI-CN and NaHSO_3_ was obtained, with a high value of, 8.2 × 10^−2^ s^−1^.

**Fig. 8:**
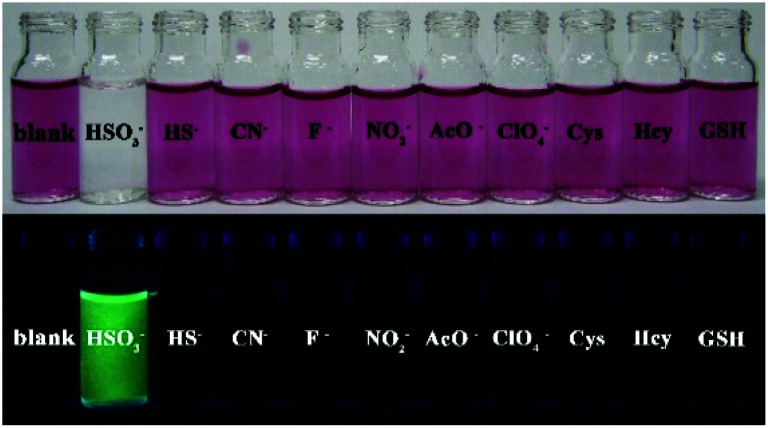
Photos for color change (upper) and fluorescence (bottom) of PI-CN (15 μM) before and after the addition of 1 equiv. of various species in EtOH/PBS (v/v 1 : 3, pH 7.4) incubation for 12 min.

The sensing behavior can be easily observed by the naked eyes from both the color change and fluorescence emission of solutions. As shown in Fig. 8, the PI-CN solutions displayed color changes under daylight and green fluorescence under a portable UV lamp only to HSO_3_^−^. Hence, PI-CN reveals a high selectivity for HSO_3_^−^ over other relevant species. The sensing reaction PI-CN with HSO_3_^−^ seems the fastest, namely, PI-CN has the highest reactivity among the four probes. Hence, other three probes should also have high selectivity for HSO_3_^−^ over other relevant species.

### PI-CN in films and test papers for detection of HSO_3_^−^/SO_3_^2−^

**Fig. 9:**
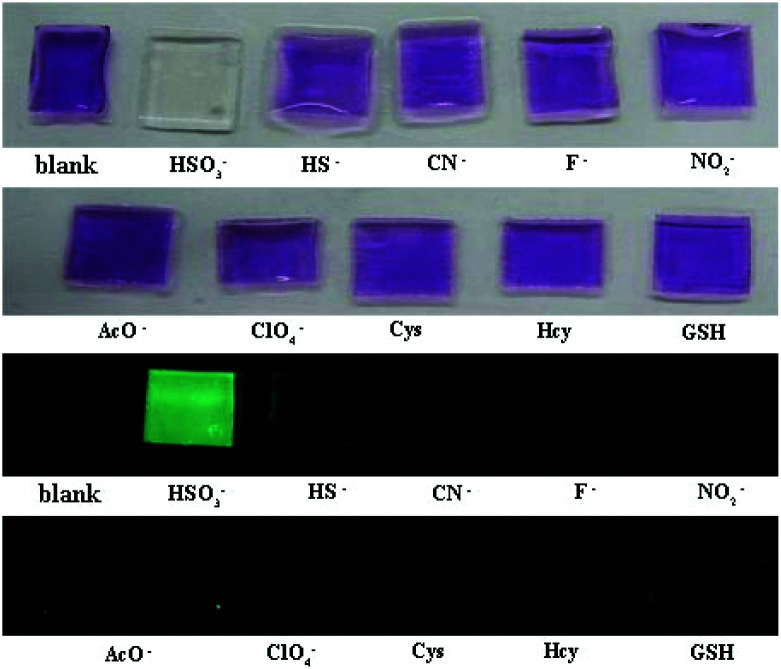
Photos for the color (upper) and the fluorescence (bottom) of films containing PI-CN before and after the addition of various species after 3 min.

**Fig. 10:**
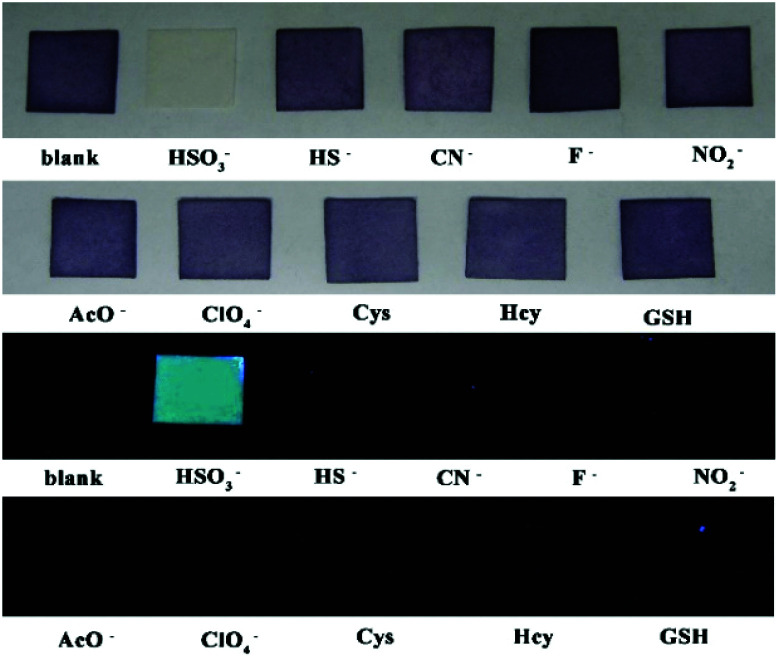
Photos for the color (upper) and the fluorescence (bottom) of filter paper containing PI-CN before and after the addition of various species after 3 min.

The film containing PI-CN was prepared to detect HSO_3_^−^/SO_3_^2−^. When the films were dipped in solutions of various species, only HSO_3_^−^ caused significant optical and fluorescent changes. The color change of the film from pink to colorless under daylight and green fluorescence under a portable UV lamp were observed, shown in Fig. 9. More easily, test papers with PI-CN were prepared with filter paper immersed PI-CN solution, then dried in air and afforded pink color test papers. The test papers were dipped in solutions of various analytes. As shown in Fig. 10, a similar color change under daylight and fluorescence emission under a portable UV lamp were observed. Because of direct observation by naked eyes, the test paper is more expediently and easier for detecting HSO_3_^−^/SO_3_^2−^.

### pH effects and MTT analysis

To assess the function of PI-CN under physiological conditions, the absorption and fluorescence spectra of PI-CN before and after addition of HSO_3_^−^ were recorded under different pH values. The pH-dependent absorption and fluorescence responses of PI-CN to HSO_3_^−^ reveal a remarkable change of absorbance and significant fluorescence enhancements at 499 nm under physiological conditions (pH 6–9) (Fig. S4†). These results indicate that PI-CN could be used as fluorescent probe in a biological system

**Fig. 11:**
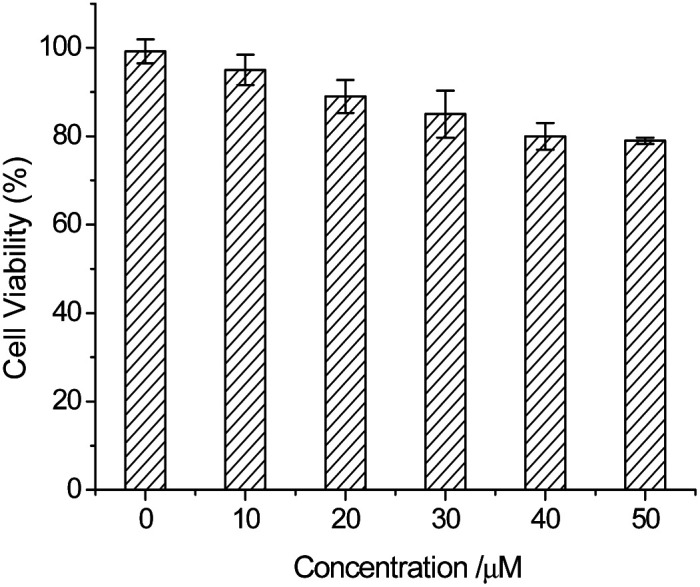
MTT assay of HeLa cells in the presence of different concentrations of PI-CN.

In order to detect HSO_3_^−^/SO_3_^2−^ in living cells, a MTT analysis was performed to assess the cytotoxicity of the probe. In the MTT assays, HeLa cells were dealt with PI-CN at different concentrations from 10 to 50 μM for 24 h. The results show low toxicity to cultured cells under the experimental condition, and the cell viability is up to 80% for PI-CN at 50 μM (Fig. 11). This result shows that PI-CN is of very low cytotoxicity.

### Cell imaging

**Fig. 12:**
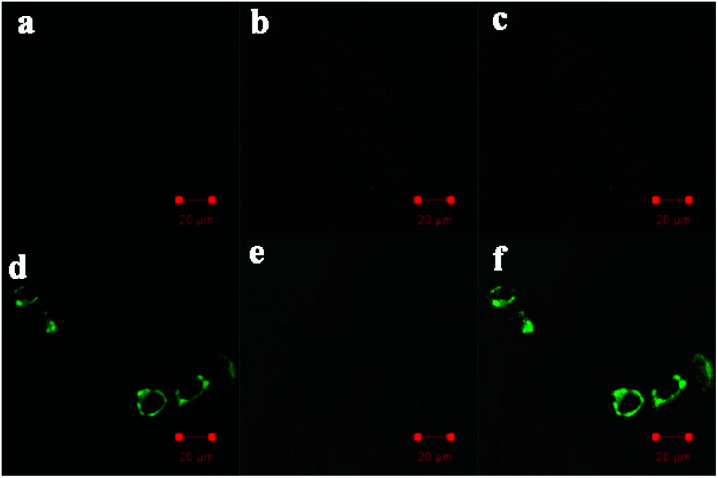
Confocal fluorescence images of HeLa cells incubated with 10 μM PI-CN for 30 min (a–c) and then 0.1 mM HSO_3_^−^ for 30 min (d–f). Images (a and d) were acquired using 405 nm excitation and emission channels of 476–526 nm. Images (b and e) were acquired from bright field, and images (c and f) were overlapped by (a) and (b) or (d) and (e).

Finally, the probe PI-CN was utilized for imaging in HeLa cells. HeLa cells were seeded on a 24-well plate in a culture medium for 24 h. The HeLa cells were incubated with PI-CN (10 μM) for 30 min, followed by PBS washing for three times. Confocal fluorescence exhibited no emission for the cells incubated with the probe at respective channels with excitation at 405 nm (Fig. 12a–c). This implies that PI-CN has no response toward biomolecules such as biothiols in living cells. In contrast, after further incubated with 0.1 mM NaHSO_3_ for 30 min, the HeLa cells emit bright green fluorescence (Fig. 12d–f). It shows that the probe PI-CN is a cell membrane permeable fluorescent probe and can be achieved to detect HSO_3_^−^/SO_3_^2−^ in living cells.

## Conclusions

In conclusion, four 7-substituted phenothiazine-hemicyanine dyes (PI-CN, PI-Br, PI-H, PI-OH) were synthesized as colorimetric and fluorescent probes for the detecting of HSO_3_^−^/SO_3_^2−^ from the condensation reactions of four different substituted phenothiazine aldehydes with 1-ethyl-2,3,3-trimethylindolium iodide. All probes can sensitively respond HSO_3_^−^/SO_3_^2−^*via* a Michael addition to cause remarkable color change. The sensing reactions displays remarkable substituent effect on fluorescence property of their sensing products. Among them, PI-CN can detect HSO_3_^−^/SO_3_^2−^ fast as both a colorimetric and fluorescent probe with the lowest detection limit (7.5 nM). Both acylamide films and the test papers of PI-CN can detect HSO_3_^−^/SO_3_^2−^ in solutions fast and selectively, and observed conveniently by naked eyes. Furthermore, cell-imaging experiments reveal that PI-CN can detect HSO_3_^−^/SO_3_^2−^ selectively in biological environment.

## Conflicts of interest

There are no conflicts to declare.

## Supplementary Material

RA-011-D1RA06868G-s001
