# Supplementary material for: Phenothiazine and semi-cyanine based colorimetric and fluorescent probes for detection of sulfites in solutions and in living cells
Source: RSC Adv. 2021 Oct 26;11(55):34643–51. doi: 10.1039/d1ra06868g (PMC9042716; doi:10.1039/d1ra06868g)

Electronic Supplementary Material (ESI) for RSC Advances.
This journal is © The Royal Society of Chemistry 2021

Electronic Supplementary Information for

Phenothiazine and semi-cyanine based colorimetric and fluorescent probes for detection of sulfites in solutions and in living cells

Hong-Wei Chen, Hong-Cheng Xia*, O. A. Hakeim, Qin-Hua Song*

Department of Chemistry, University of Science and Technology of China, Hefei 230026, China.

Table of Contents

Table of Contents

I. Photophysical and sensing properties of four probes ...…………………………………………S2
II. Spectral response of probes to HSO_3^-/SO_3^{2-}…………..……..…………………………S2
III Mass spectra of PI-CN without and with $NaHSO_3$…………………………………….S3
IV. Measurement of detection limits……………………………………………………… S4
V. pH effects on optical response of PI-CN to HSO_3^-/SO_3^{2-}……………………………..S5
VI. NMR spectra of related compounds …………………………………………………S6-S11

* Corresponding author. E-mail address: qhsong@ustc.edu (Q.-H. Song)
* Corresponding author. E-mail address: xiahc@mail.ustc.edu.cn (H.-C. Xia)

I. Photophysical and sensing properties of four probes

Table S1. Photophysical and sensing properties of four probes

probe	$\lambda_{abs}(\varepsilon)^{a}$ /nm	$(\varepsilon)/(L \cdot mol^{-1} \cdot cm^{-1})^{a}$	λ_{em}^{b} /nm	LOD
PI-CN	518	30320	499	22 nM
PI-Br	537	28320	452	28 nM
PI-H	545	29180	455	27 nM
PI-OH	568	25846	470	37 nM

[a] absorption maxima (nm) and molar absorption coefficients
[b] emission maxima (nm)

II. Spectral response of probes to HSO_3^-/SO_3^{2-}

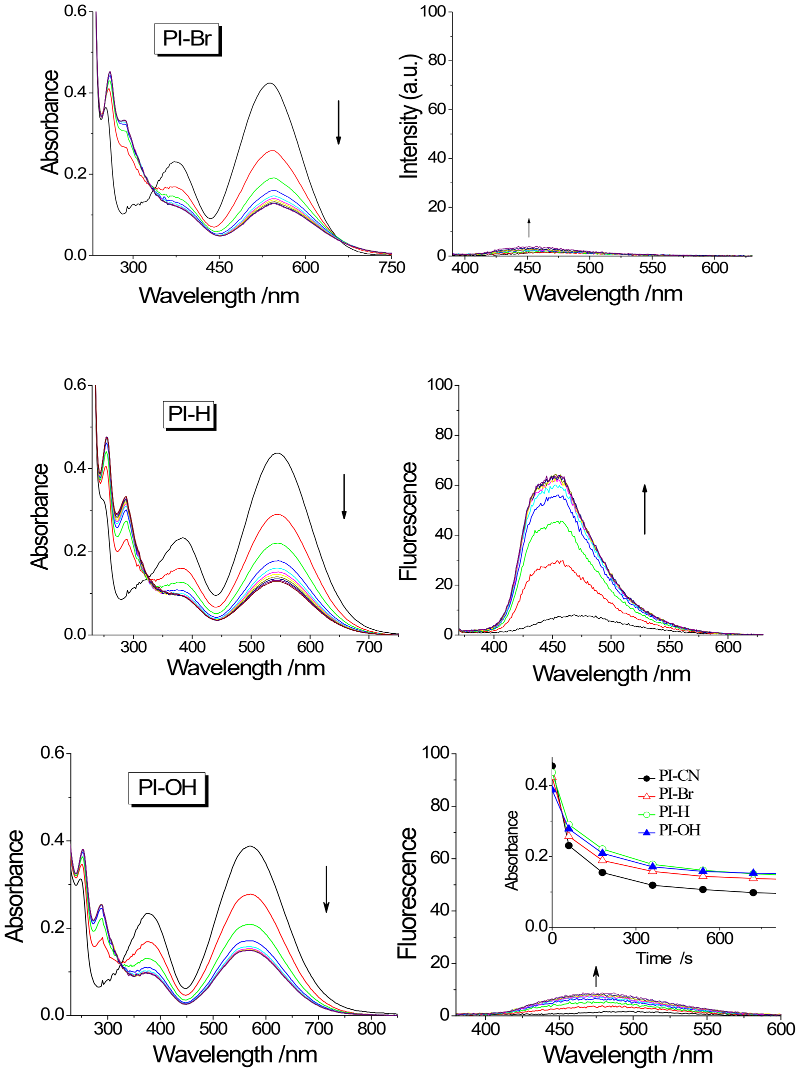

Fig. S1 Time-dependent UV/vis absorption (left) and fluorescence spectra (right) of probes (15 μM) in EtOH/PBS (v/v1:3, pH 7.4) in the presence of HSO_3^- (1.0 equiv.) recorded at 0-30 min, excitation at 320 nm. Inset of PI-OH: plots of absorption maxima of probes *vs* time in the presence of HSO_3^- incubation for 15 min

III. Mass spectra of PI-CN without and with $NaHSO_3$

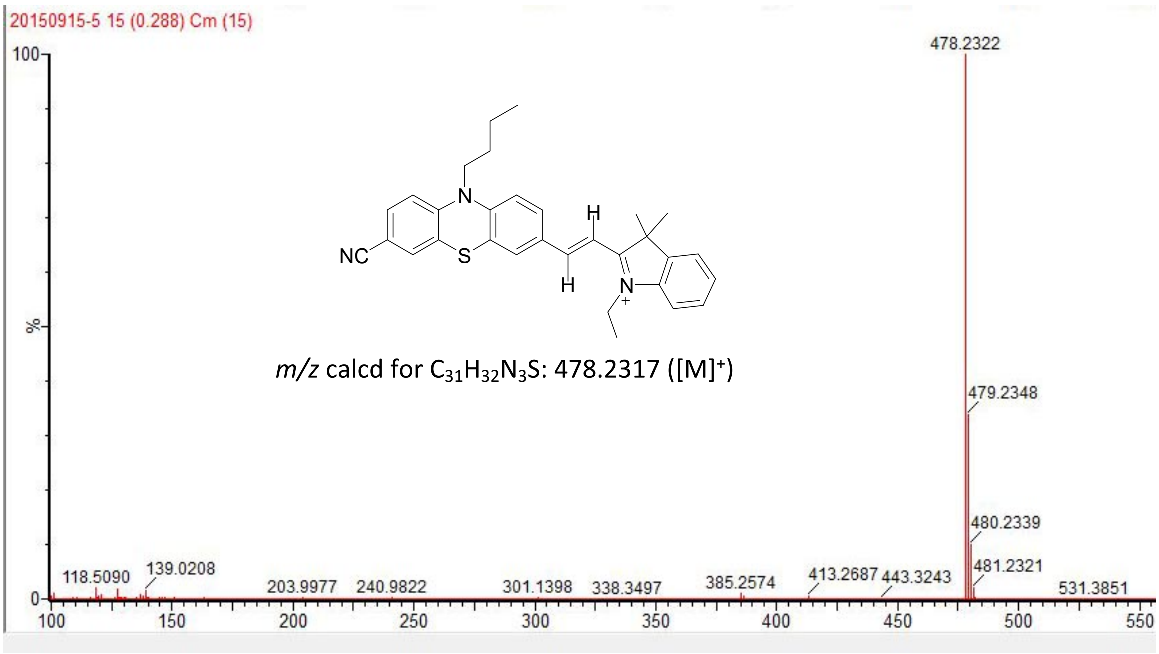

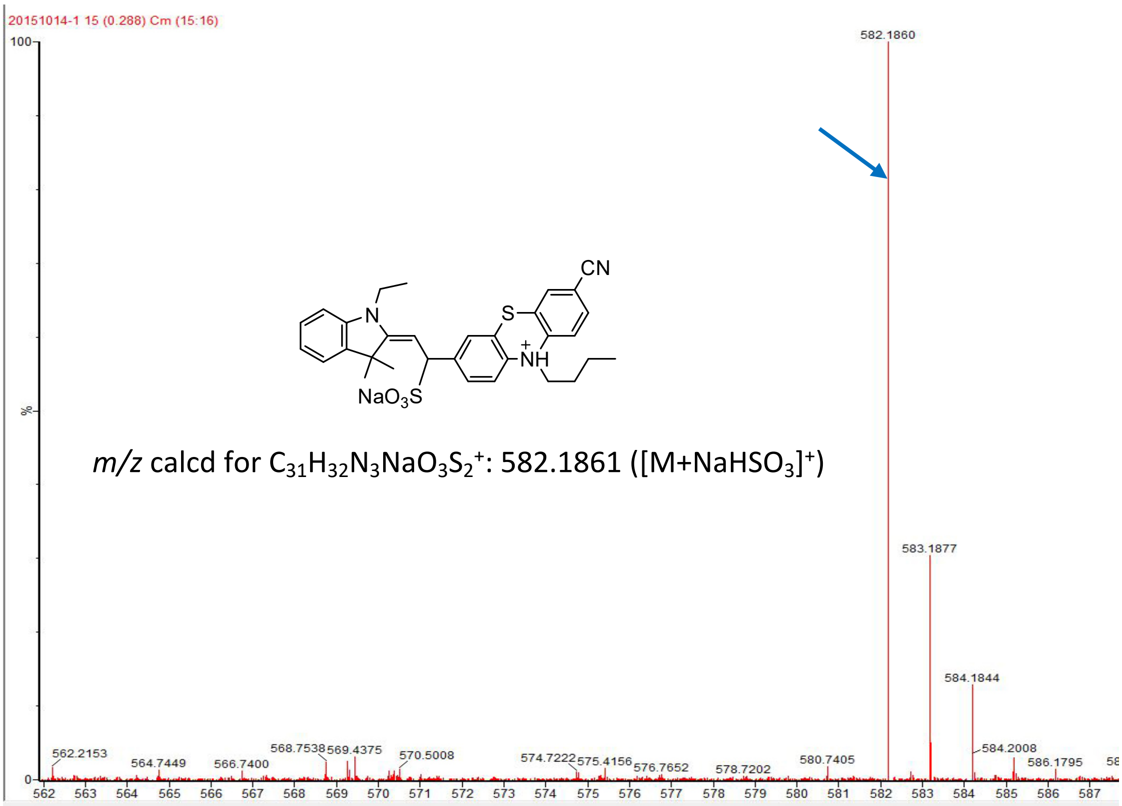

Fig. S2 High-resolution MS of probe PI-CN (upper) and the mixture of PI-CN+$NaHSO_3$ (bottom).

IV. Measurements of detection limits

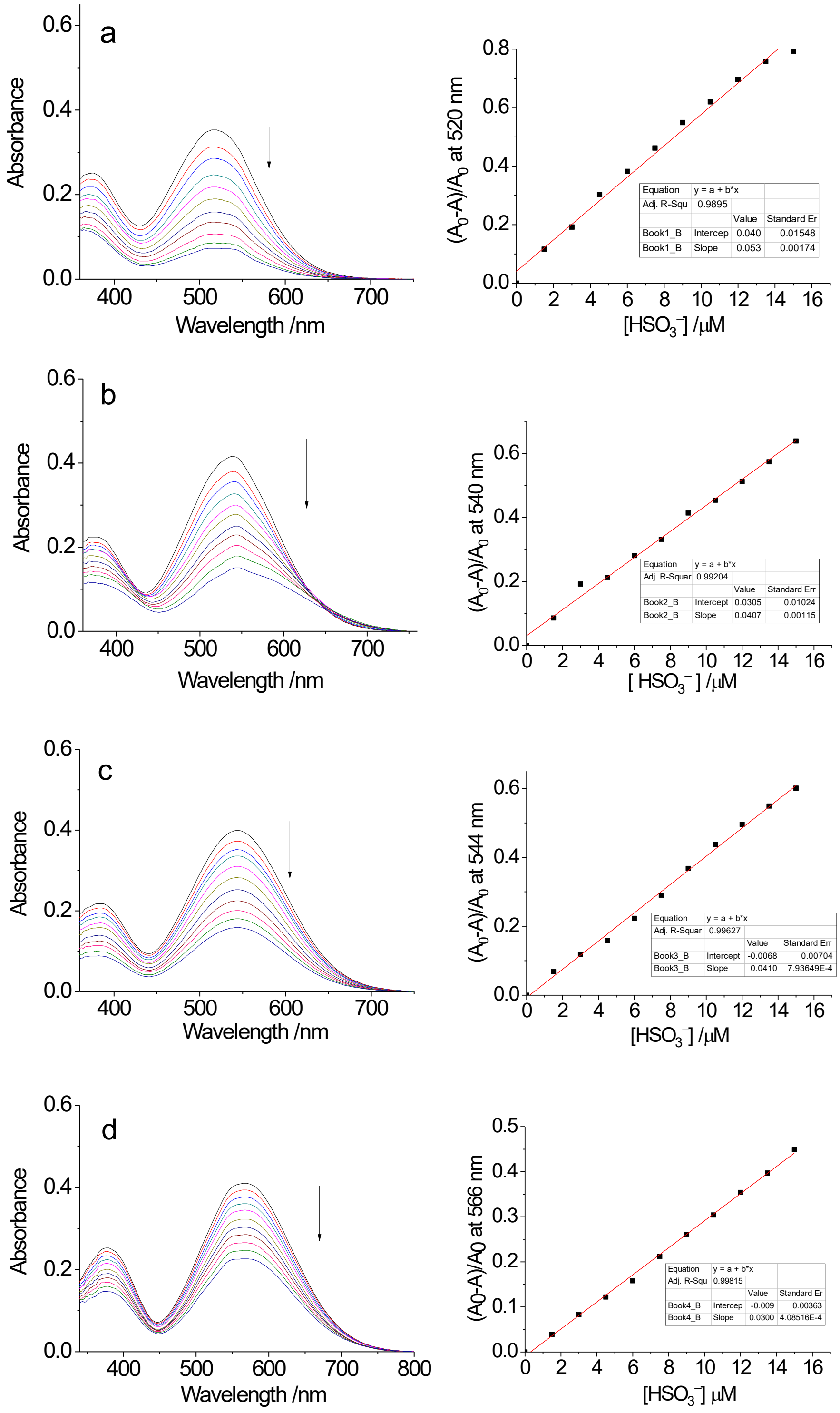

Figure S3. UV/vis absorption PI-CN (a), PI-Br (b), PI-H (c) and PI-OH (d) in EtOH/PBS (v/v1:3, pH 7.4) with titration of various amounts of HSO_3^- (0–15 μM), and the corresponding linear correlation between the absorbance toward concentrations of HSO_3^-.

V. pH effects on optical response of PI-CN to HSO_3^-/SO_3^{2-}

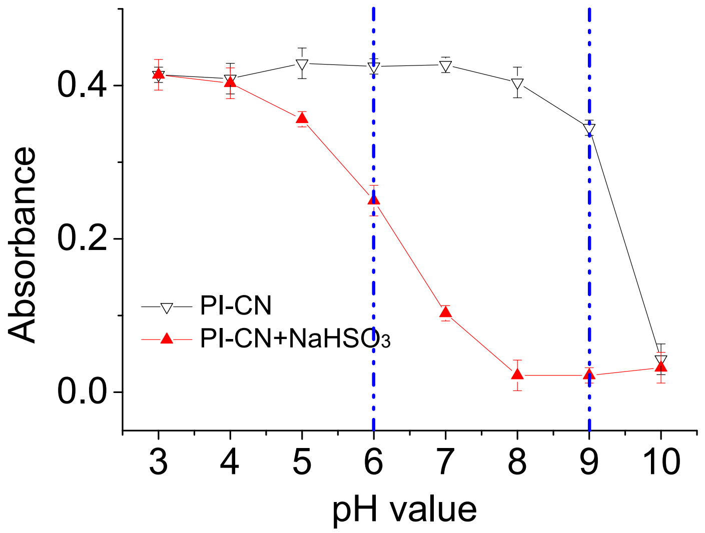

Figure S4a. Plots of absorbance at 520 nm to pH values for 15μM PI-CN solutions (EtOH/PBS v/v 1:3) before (black) and after (red) the addition of 15 μM HSO_3^-.

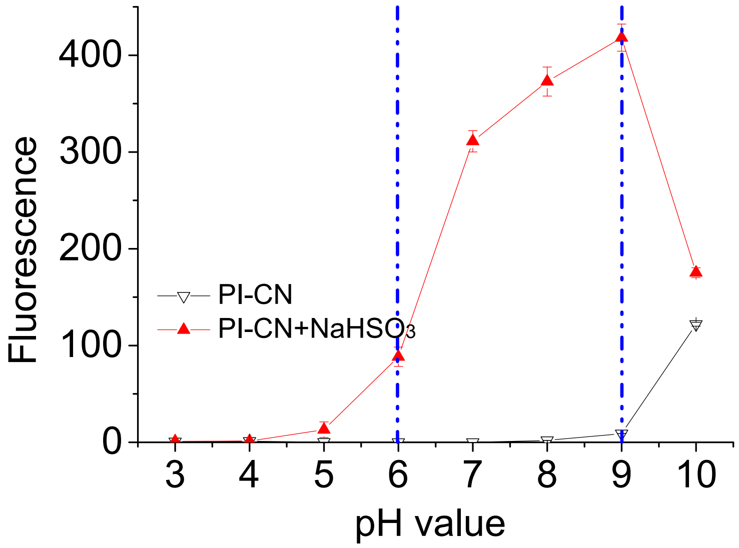

Figure S4b. Plots of fluorescence intensity at 499 nm to pH values for 15μM PI-CN solutions (EtOH/PBS v/v 1:3) before and after the addition of 15 μM HSO_3^-.

IV. NMR spectra of related compounds

^{1}H NMR of compound **3**.

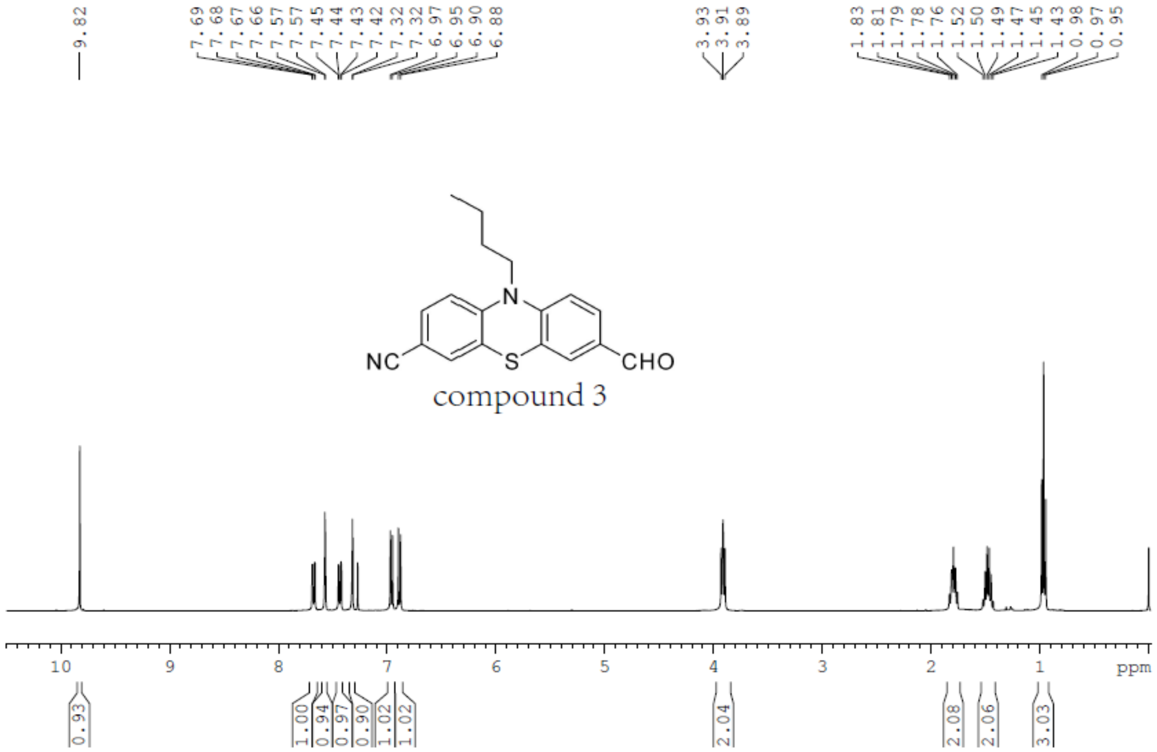

^{13}C NMR of compound **3**.

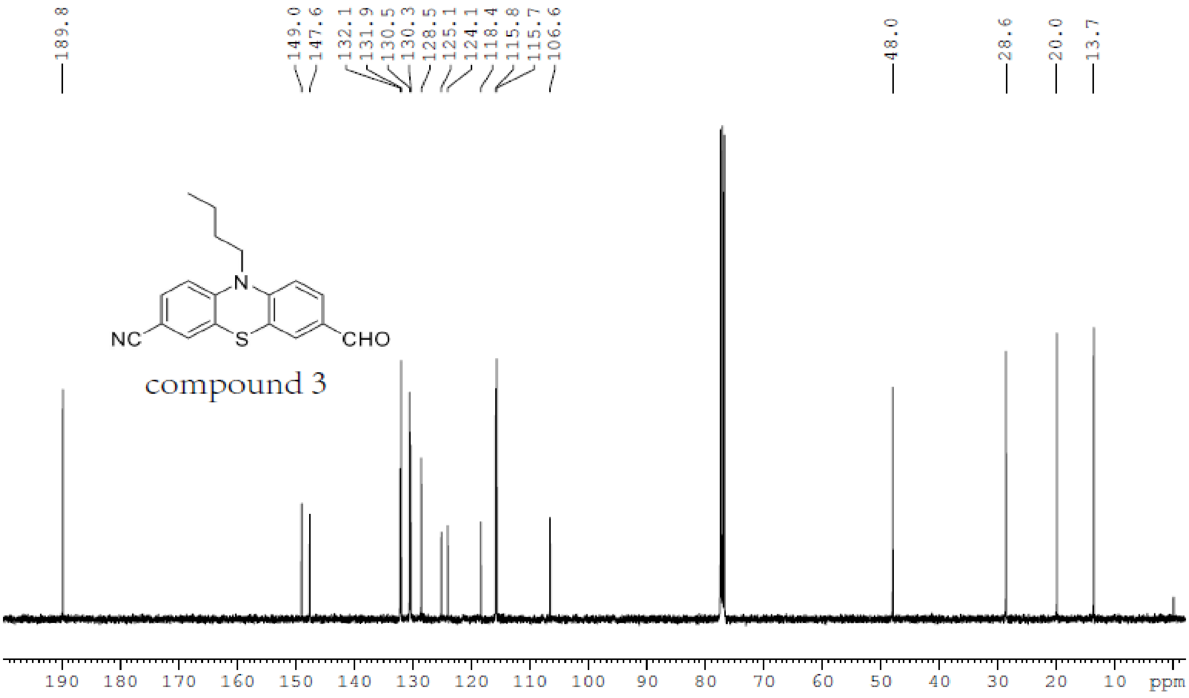

^{1}H NMR of compound **4**.

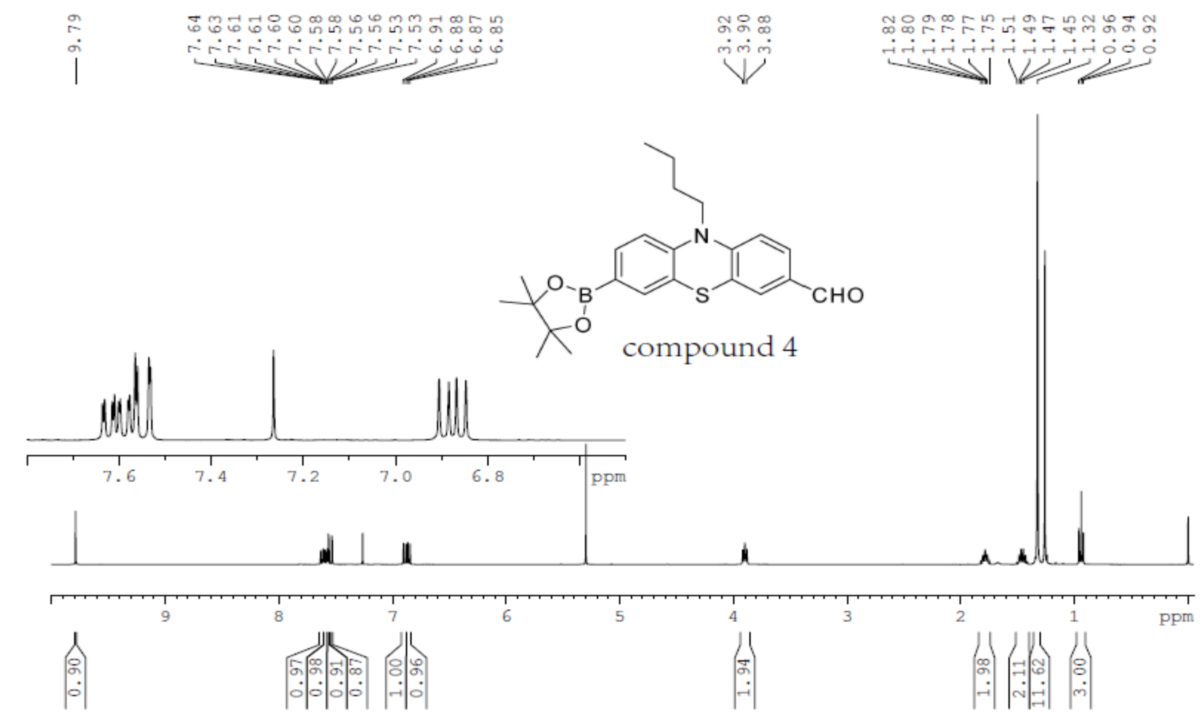

^{13}C NMR of compound **4**.

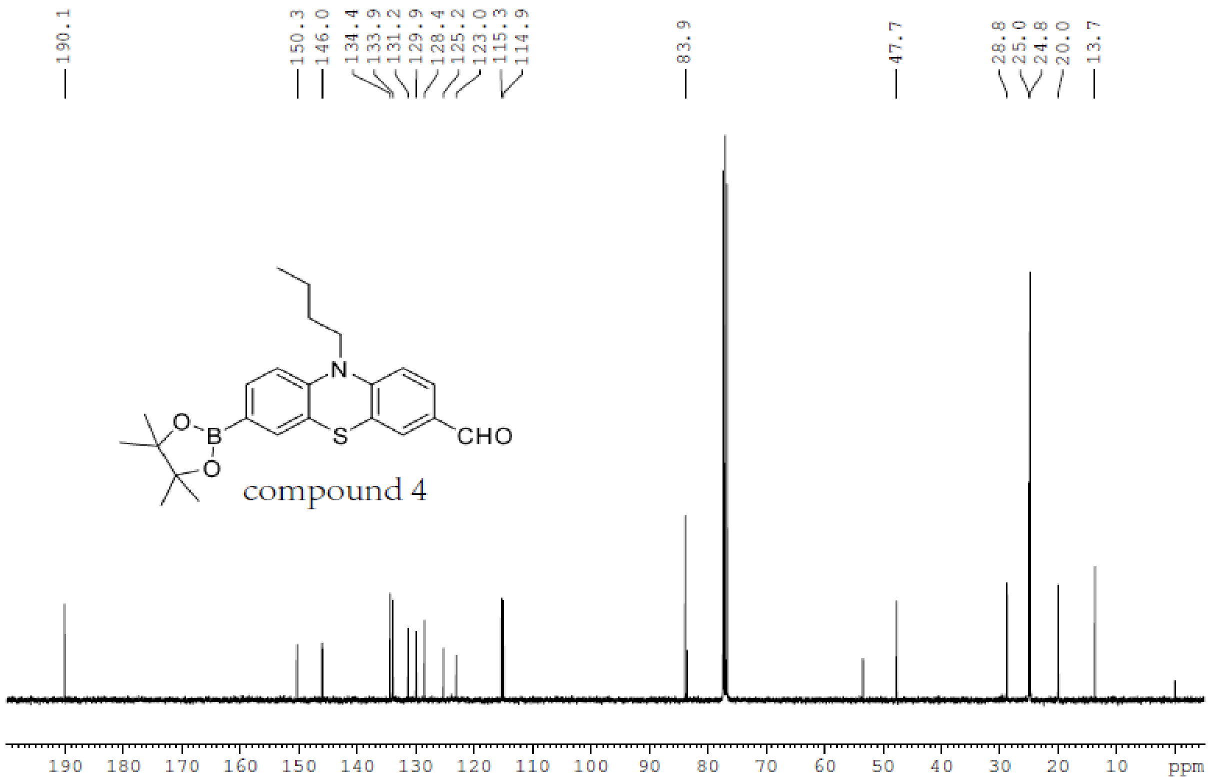

^{1}H NMR of compound **5**.

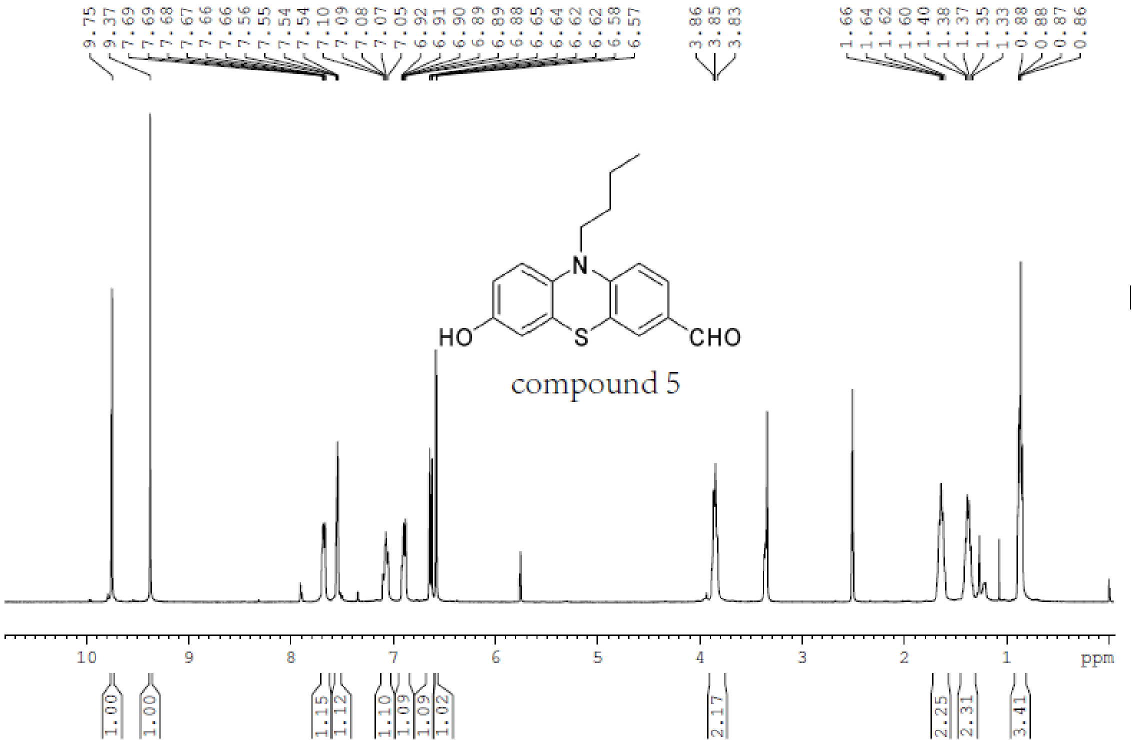

^{13}C NMR of compound **5**.

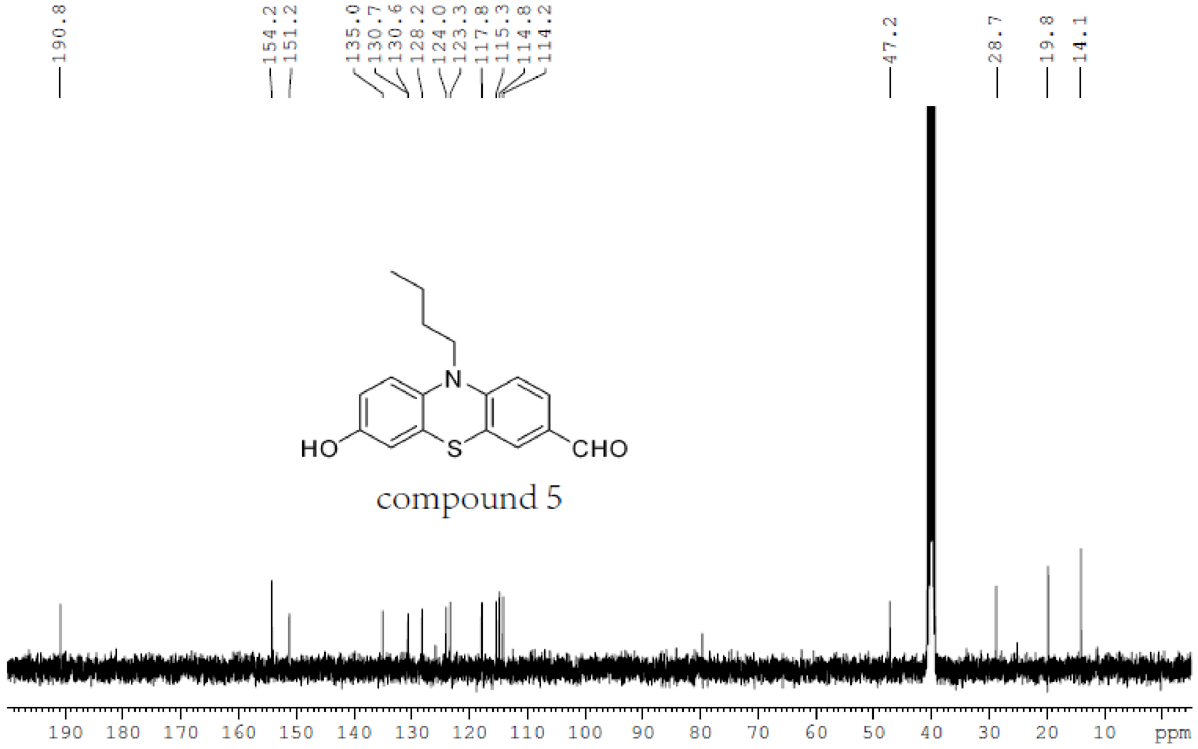

^{1}H NMR of PI-CN.

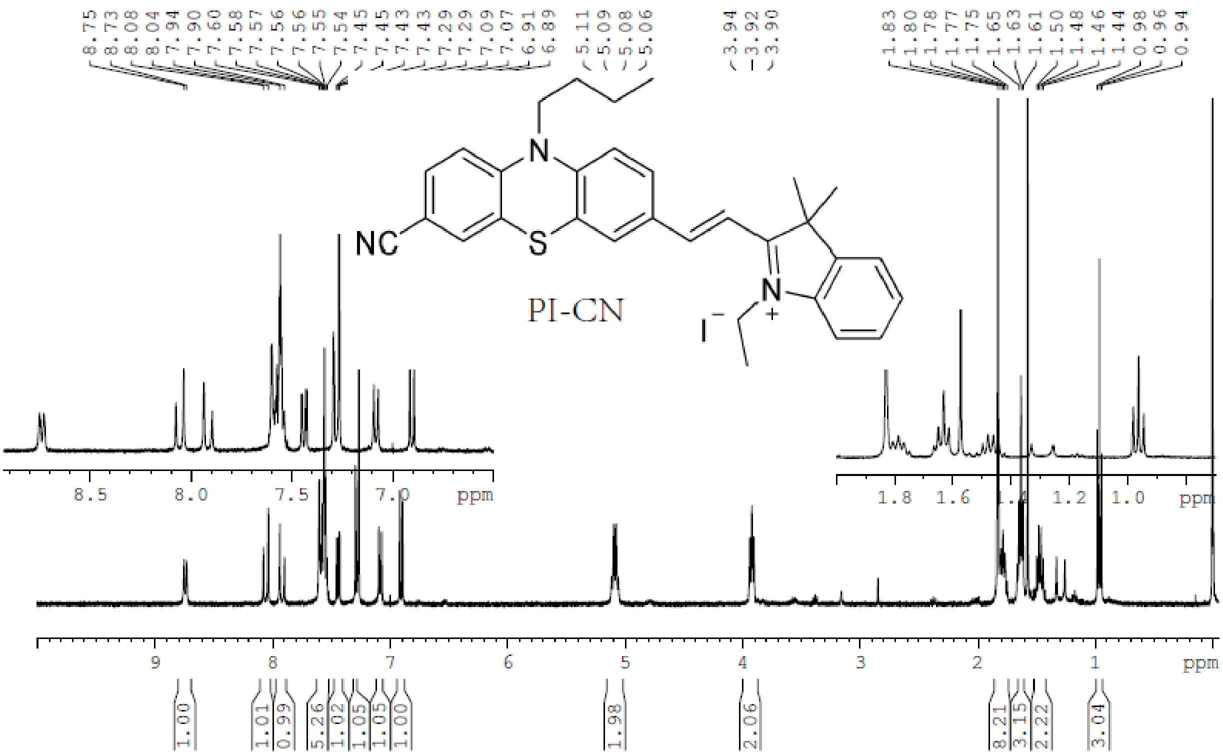

^{13}C NMR of PI-CN.

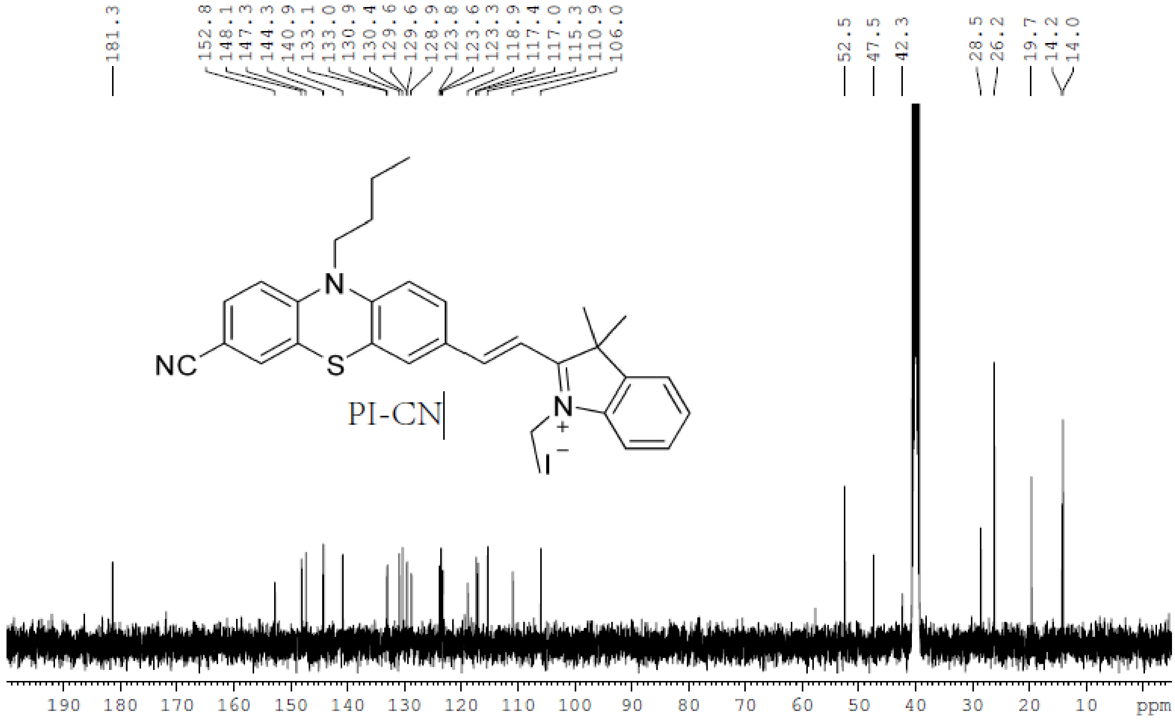

^{1}H NMR of PI-Br.

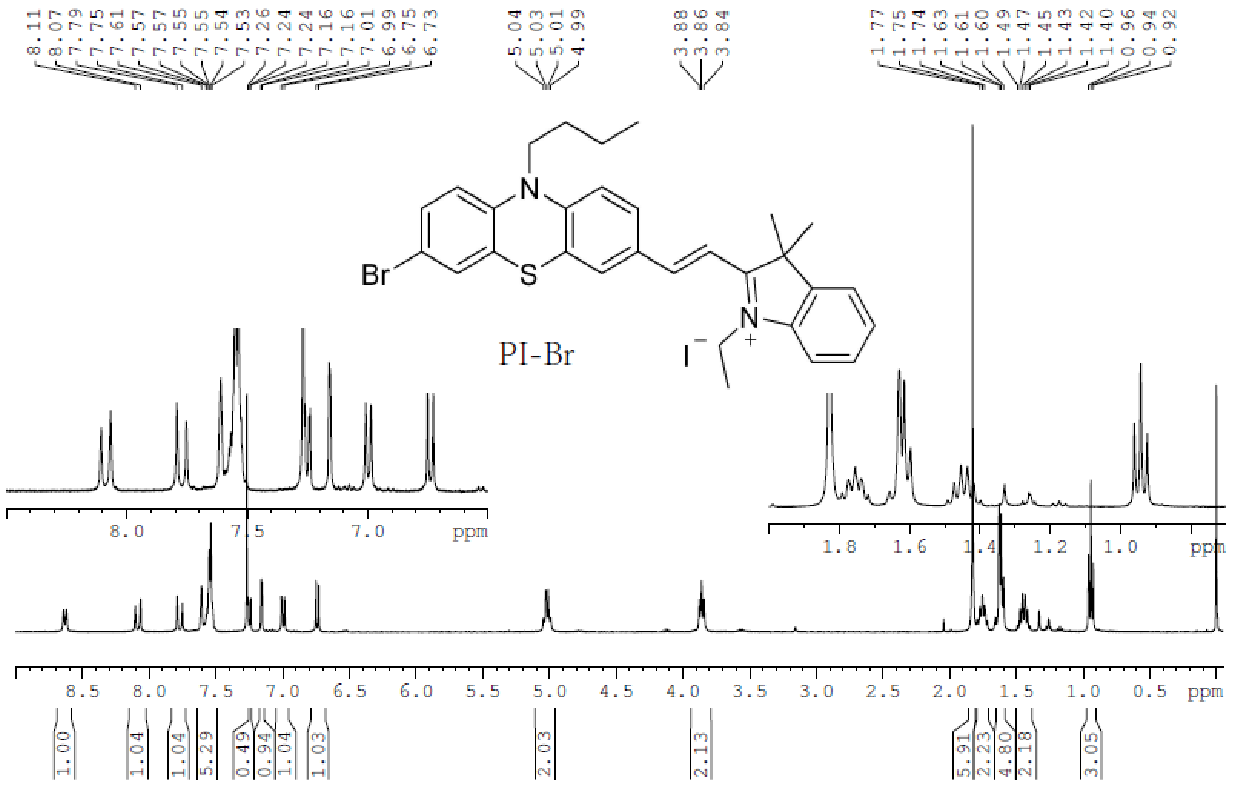

^{13}C NMR of PI-Br.

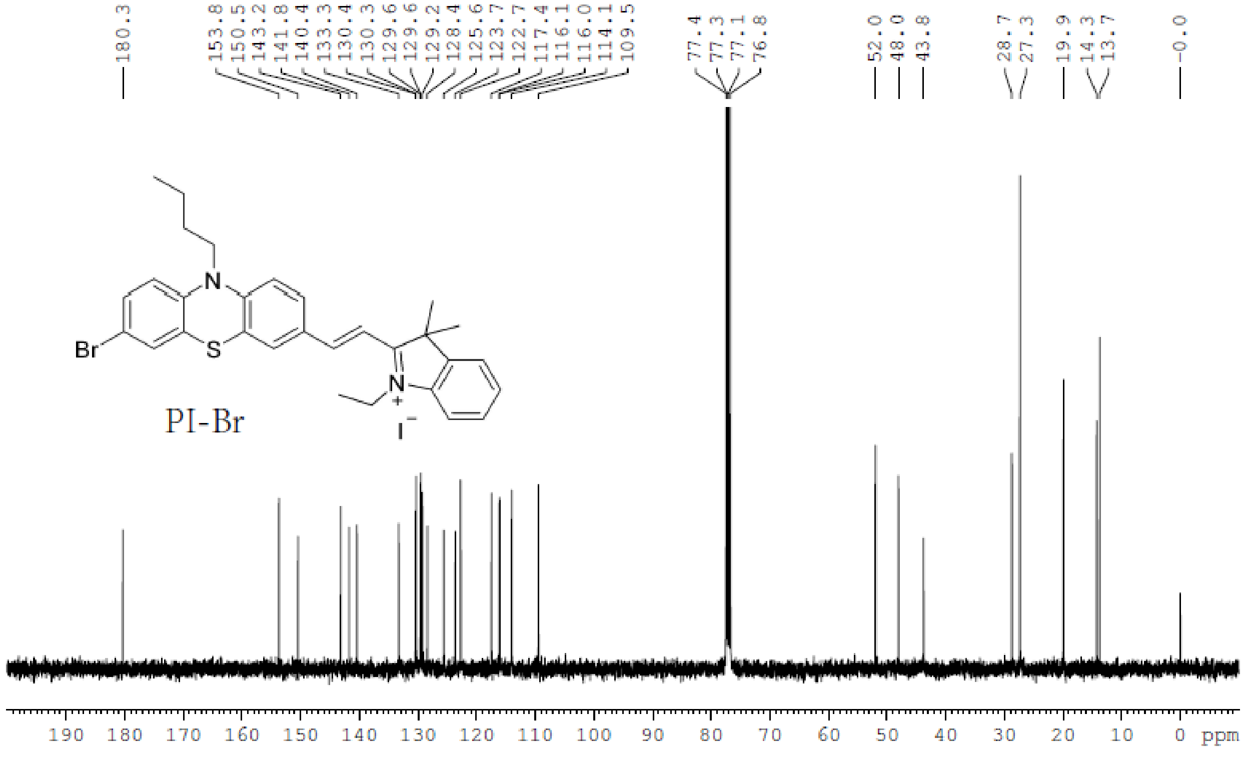

^{1}H NMR of PI-H.

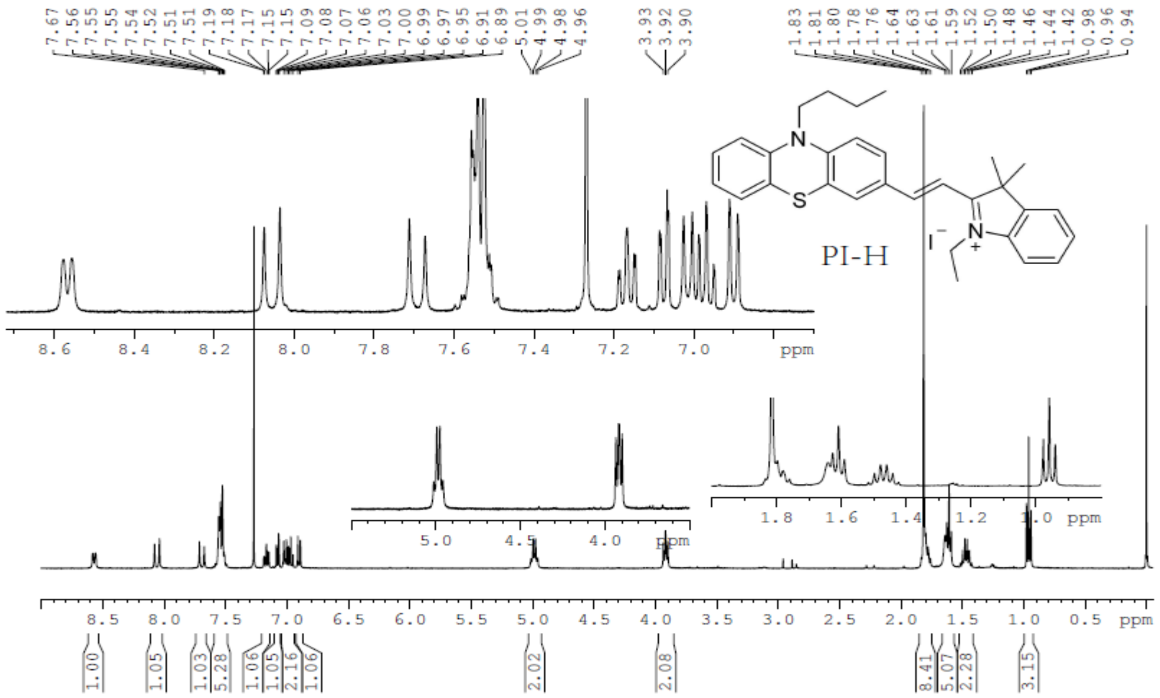

^{13}C NMR of PI-H.

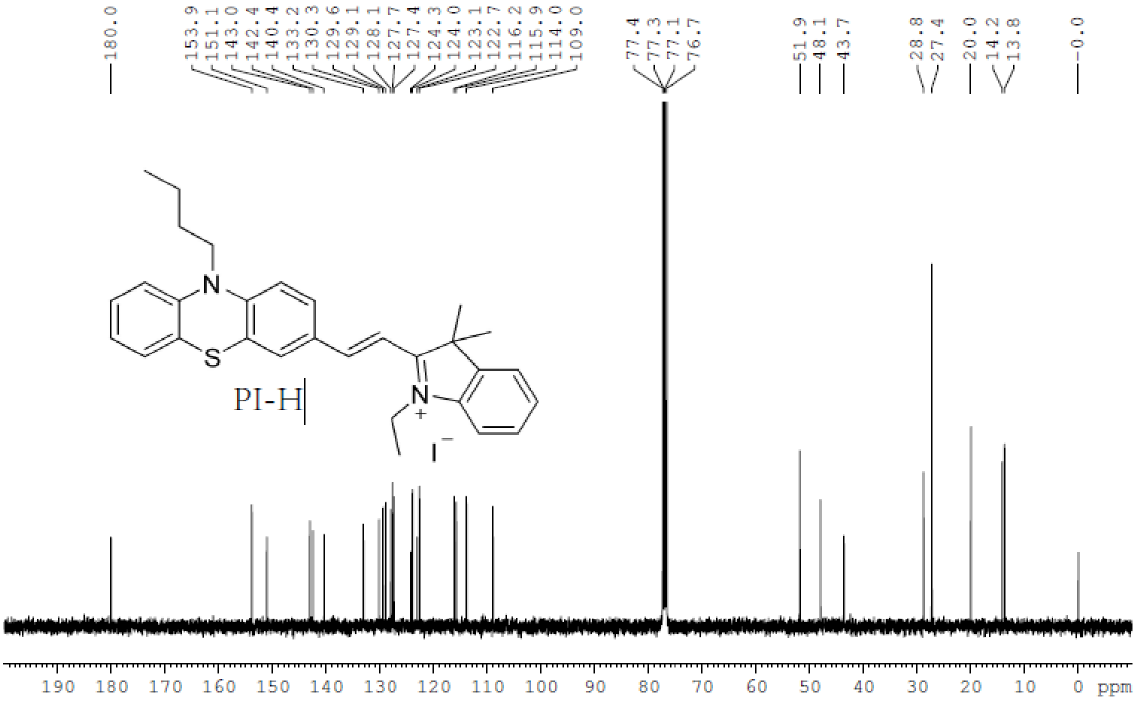

^{1}H NMR of PI-OH.

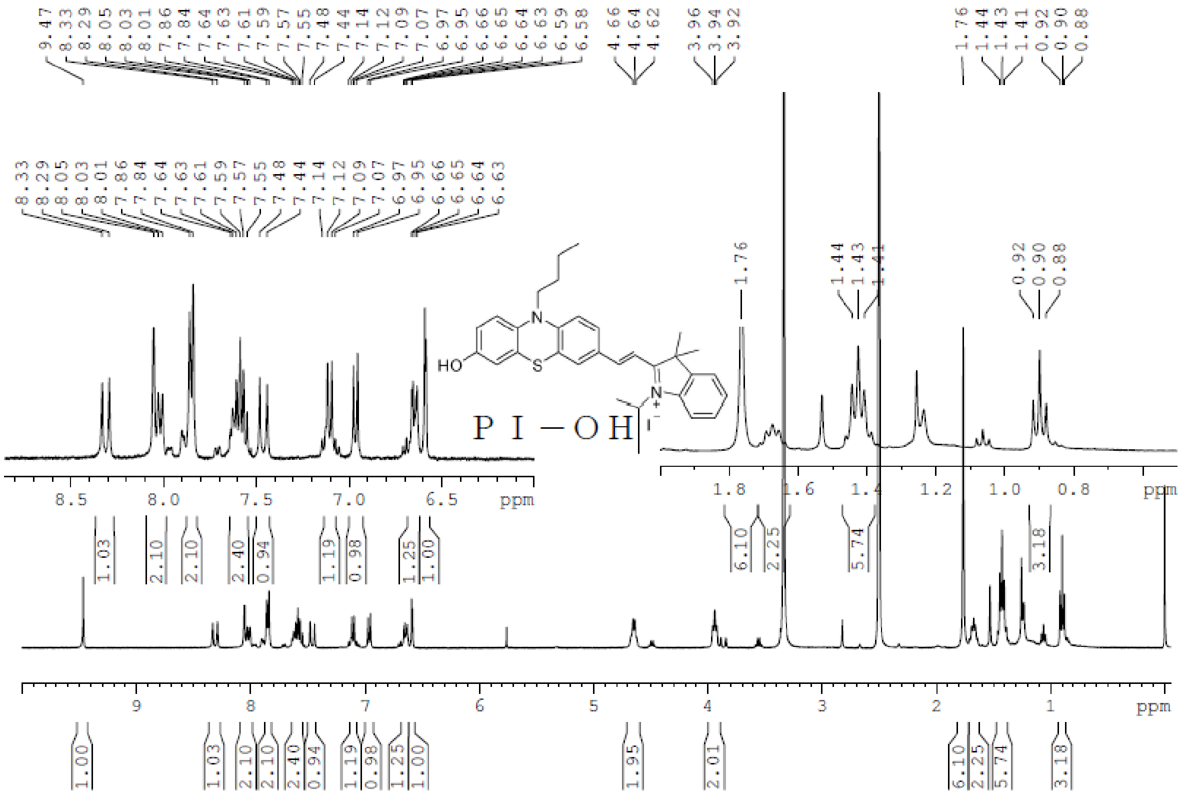

^{13}C NMR of PI-OH

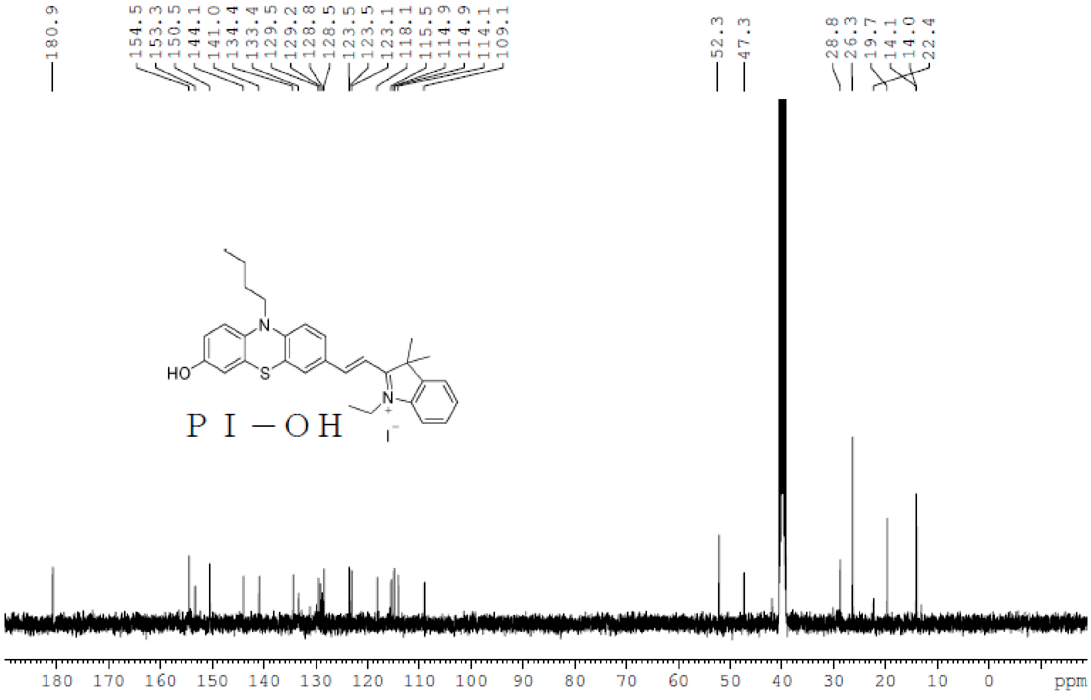

Supplement: RA-011-D1RA06868G-s001 [file RA-011-D1RA06868G-s001.pdf]
